# The effect of the modified basic package of oral care on adolescent dental caries status in Zambia; a cluster randomized trial

**DOI:** 10.3389/froh.2025.1542337

**Published:** 2025-05-07

**Authors:** Severine N. Anthony, Hawa S. Mbawalla, Febronia K. Kahabuka, Seter Siziya, Anne N. Åstrøm

**Affiliations:** ^1^ Department of Dental Clinical Sciences, Michael Chilufya Sata School of Medicine, The Copperbelt University, Ndola, Zambia; ^2^ Department of Orthodontics, Paedodontics and Community Dentistry, School of Dentistry, Muhimbili University of Health and Allied Sciences, Dar es Salaam, Tanzania; ^3^ Department of Clinical Dentistry, Faculty of Medicine and Dentistry, University of Bergen, Bergen, Norway; ^4^ Department of Public Health, Michael Chilufya Sata School of Medicine, The Copperbelt University, Ndola, Zambia

**Keywords:** effect, modified, basic package of oral care, dental caries, adolescents

## Abstract

**Background:**

The Basic Package of Oral Care (BPOC) was developed by the World Health Organization (WHO) in order to improve oral health care worldwide, yet evidence of its effectiveness is scarce. This study primarily assessed the outcome of applying modified BPOC on dental caries prevalence and secondarily on knowledge and behaviors related to dental caries among adolescents in Copperbelt Province, Zambia.

**Methods:**

A parallel arms cluster randomized field trial including 22 public secondary schools (11-intevention, 11-control) was carried out between January 2021 and March 2023 in Copperbelt Province, Zambia. A validated questionnaire collected data with respect to socio-demographics, knowledge, and dental caries-related behaviors. The caries assessment spectrum and treatment (CAST) instrument was used to examine the spectrum of carious lesions during the baseline, and the follow-up data collection phases. The 1st and 2nd follow up exams were conducted at 18 and 24 months after baseline, respectively. The intervention group received a six-month duration modified BPOC intervention while, the control group continued with their routine oral self-care. The analysis was based on the intention-to-treat protocol using generalized estimating equations (GEE), and the results were reported as OR (95% CI).

**Results:**

Out of 1,794 participants at baseline, 1,690 (94.2%) and 1,597 (89.0%) were examined at the 1st and 2nd follow up intervals, respectively. Dental caries models showed significant interaction at 18 and 24 months [OR (95% CI) = 0.7 (0.6, 0.8), *p* < 0.001]. Adequate knowledge and use of fluoridated toothpaste models were the only secondary outcomes with significant interactions at 18 and 24 months follow up. Stratified analysis at 18 and 24 months showed that the intervention group had better outcomes for adequate knowledge, use of fluoridated toothpaste and dental caries.

**Conclusion:**

The modified BPOC was effective in reducing the prevalence of dental caries, improving knowledge on dental caries, and increasing the frequency of using fluoridated toothpaste among Zambian adolescents.

**Clinical Trial Registration:**

[https://pactr.samrc.ac.za/TrialDisplay.aspx?TrialID=24046], identifier [PACTR202210624926299].

## 1 Introduction

Despite dental caries being preventable, its prevalence continues to rise, especially in low- and middle-income countries (1). A large proportion of dental caries remains untreated among adolescents, thus negatively impacting their general health, wellbeing, and quality of life (2–4). Studies that include 10–19 years old school children in Zambia report dental caries prevalence's ranging from 11.4%–20% (5, 6). In the country currently implemented school-based interventions include dental professional-led oral health education and outreach services organized by dental training schools, the ministry of health and non-governmental organizations. The effectiveness of the interventions remains unknown as the findings are unpublished. However, one study reported the effect of outreach services at rural health posts among all age groups which were effective in reducing untreated dental caries and relieving pain (7).The World Health Organization (WHO) recommends reducing the burden of oral diseases by emphasizing comprehensive system changes to shift from traditional curative approaches towards preventive approaches such as the application of the basic package of oral care (BPOC) (8).

The WHO recommend use of preventive interventions that are acceptable, practicable, and affordable to most disadvantaged communities such as BPOC (8). The strategy is critical to fulfilling the core goal of universal health coverage (UHC), which is providing better health services to all (9). It is also in line with the World Health Organization's (WHO) recommendation of reorienting traditional curative-oriented oral health services towards approaches that adhere to the principles of primary health care (PHC) (8, 10).

BPOC consists of three packages: Oral Urgent Treatment (OUT), Atraumatic Restorative Treatment (ART), and Affordable Fluoride Toothpaste (AFT). OUT is achieved by offering simple extraction of teeth beyond a restorable state and referral of complicated cases. ART is provided by restoring teeth with reversible pulpitis on cavities, which can be accessed by hand instruments only without the need for rotary instruments, and then restored using glass ionomer cement. AFT can be achieved through regulatory strategies such as lowering or abolishing taxes on the items with the goal of reducing their market prices (8). The World Health Organization recommends tailoring BPOC to the local environment and evaluating its effectiveness in improving oral health needs in various locations around the world (8).

Although the effectiveness of the individual components of BPOC has been demonstrated in previous studies, the evaluation of its overall success as a package has received little attention. A study in Cambodia demonstrated success in increasing extractions and ART-restored teeth at follow-up following BPOC intervention (11). Another study reported success in reducing the incidence of early childhood caries among children of women who received BPOC during prenatal and postnatal periods (12). To the best of our knowledge, only two studies have reported the effectiveness of BPOC as a package and this is the first study reporting its effects on dental caries among adolescents in Sub Saharan Africa cultural context. We aimed to assess the outcome of applying the modified basic package of oral care to dental caries status among adolescents in Copperbelt Province, Zambia. The secondary objectives of the study were to determine the outcome of modified BPOC on knowledge and behaviors related to dental caries.

The hypothesis tested for the primary objective was that no difference in dental caries status between the intervention and control groups would be observed at follow ups. The hypotheses for the secondary objectives were that there would be no difference between the intervention and control groups at follow-ups with regards to adequate knowledge and consuming sugary drinks and foods less than five times per day. The study also hypothesized no difference between groups in using fluoridated toothpaste twice or more per day and visiting a dentist at least once in the previous year.

## 2 Materials and methods

### 2.1 Study design

The study used a parallel two-arm cluster randomized controlled field trial design with one control group and one intervention group (allocation ratio 1:1). The protocol received ethical approval from three institutions: the study site (Tropical Diseases Research Centre, Zambia, IRB 00002911, FWA 00003729) and training institutions (MUHAS Institutional Review Board, Tanzania, P. MUHAS-REC-4-2020-208), and the Regional Ethical Committee Vest 191836, Norway. The National Health Research Authority (permit number, NHRA00005/16/11/2020), provincial health directorate, and district education board secretaries granted permission to conduct the research. Written informed consents were obtained from parents or guardians, and participants were requested to sign a written assent form. The trial was registered retrospectively by the Pan African Clinical Trial Registry (PACTR202210624926299), available at https://pactr.samrc.ac.za/TrialDisplay.aspx?TrialID=24046. Regrettably, the trial was registered after its commencement due to frequent changes in standard operating procedures (SOPs) for conducting research, treatment of dental patients, and the school calendar during the COVID-19 pandemic in Zambia. We registered the trial on March 15, 2022, at the Economic Association Registry for Randomized Controlled Trials, which is accessible at https://www.socialscienceregistry.org/trials/8973 as soon as we got assurance on SOPs and the school calendar. We thereafter registered the trial at the Pan African Clinical Trial Registry, which is one of the World Health Organization's recommended clinical trial registries. The registration of all ongoing and related trials for this intervention has been confirmed by the authors. The study was conducted in accordance with the principles of the Declaration of Helsinki and reported according to Consolidated Standards of Reporting Trials (CONSORT) (Supplementary material S1) (13, 14). The enrollment of study participants and baseline data collection were done between January and May 2021, followed by instituting modified BPOC intervention in the intervention group from June to November 2021. The first follow up data collection was done between June and July 2022, while the second follow up was accomplished between February and March 2023. A detailed schedule of study activities is provided as Supplementary material S2. The number of follow-ups was reduced as soon as the trial began from three initially intended to two due to unanticipated school closures during the COVID-19 pandemic.

### 2.2 Participants

**Figure 1:**
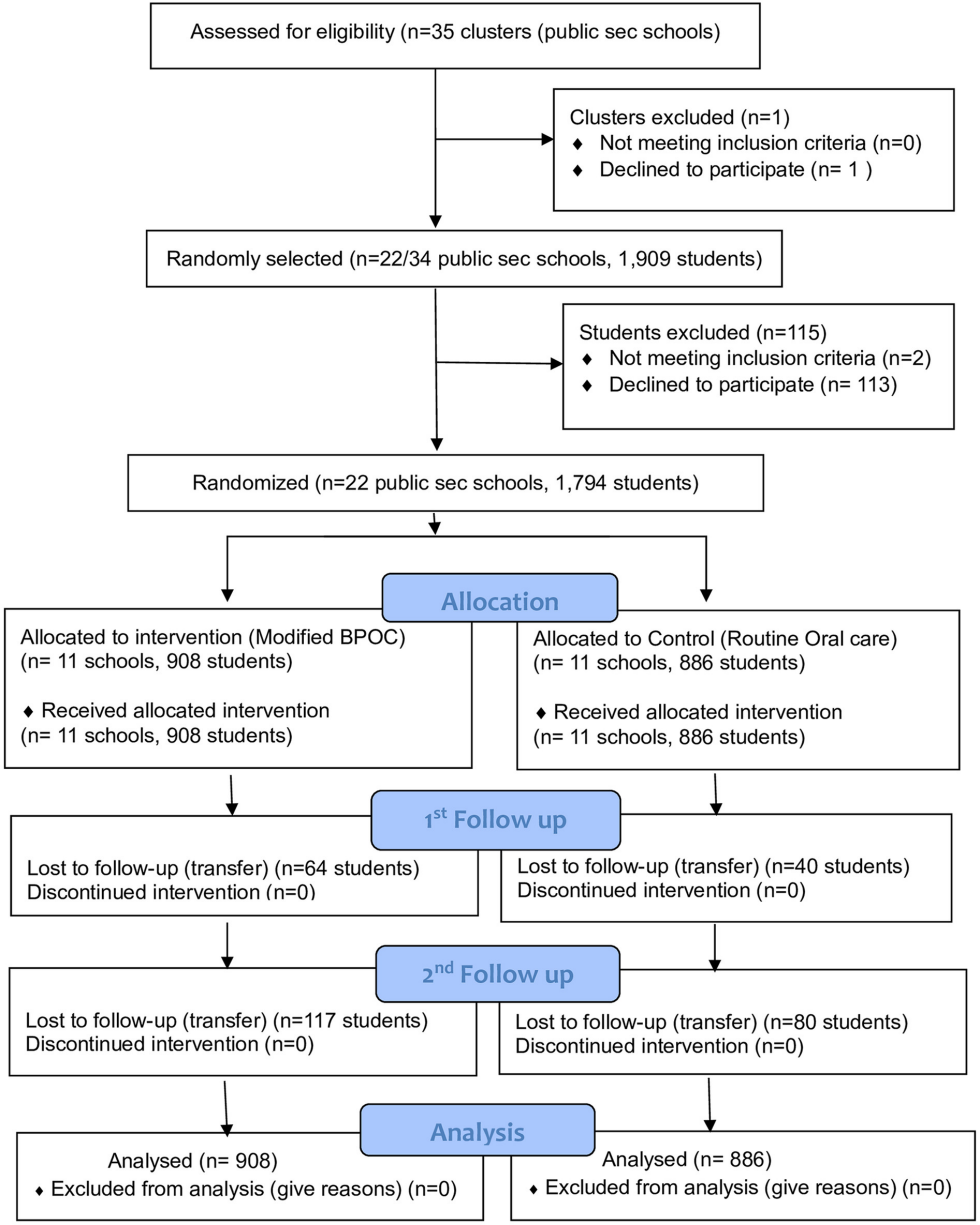
CONSORT flow chart for cluster randomised trials [cited from Campbell et al. (38)].

All adolescents in grades eight to nine studying at 35 public secondary schools in Ndola, Masaiti, and Mpongwe districts of the Copperbelt Province, Zambia, were eligible to participate (Figure 1). Out of the 35 schools, one declined to participate, resulting in 34 schools being available for random selection. Using computer-generated random numbers, 22 out of 34 schools were randomly selected. All adolescents in grades 8 and 9 who met inclusion criteria and were available during the enrollment period were invited to participate. A total of 115 students were excluded either due to being under orthodontic treatment (2) or declined to participate (113), resulting in the enrollment of 1,794 participants at baseline.

### 2.3 Intervention groups

The trial included one experimental and one control group.

#### 2.3.1 Experimental group

The participants in the experimental group received a modified World Health Organization -Basic Package of oral care (WHO-BPOC) for a period of six months in addition to their routine daily standard of oral care. The intervention adopted the Oral Urgent Treatment (OUT) and Atraumatic Restorative Treatment (ART) components of the conventional WHO-BPOC and modified Affordable Fluoride Toothpastes (AFT) component. The conventional BPOC recommends effecting the AFT component by lobbying for a reduction of prices of toothpastes through government subsidies or tax reduction, which could not be feasible in this study. In this study, the AFT component was modified by providing each participant a 500-grams pack of fluoride toothpaste once every two months and a toothbrush once every three months in order to encourage future usage of the oral care products. Peer-Led Oral Health Education (PL-OHE) was adopted as an additional component to the three components of the conventional WHO-BPOC. Oral Urgent Treatment (OUT) and Atraumatic Restorative Treatment (ART) were provided at schools by qualified dentists once in six months. OUT included; extraction of teeth beyond restorable condition at schools and referral for teeth whose treatment required a clinic set up. Whereas ART included restoring asymptomatic teeth or those in a reversible pulpitis state and with cavities accessible by hand instruments. Restorations were done by removing decayed parts of a tooth using hand instruments followed by filling the cavities with Fuji IX glass- ionomer cement. Oral health education was provided by trained peers by reading printed standardized messages (Box 1) to their classmates once every two weeks. A total of twelve peer led sessions were completed by each school in the intervention group over a period of six months. Considering the involvement of non-dental personnel, the OHE component (Box 1) was designed to be simple, objective, and easy to deliver. Periodic reminders to read the oral health messages were sent to the peer leaders every two weeks per month, through teachers assigned by the headteachers to assist the modified BPOC project organizers.

**BOX 1 Oral health messages.**

**Table:** 

1. Restrict the frequency of taking sugary foods and drinks in the diet to less than five times per day (15). 2. Brush your teeth for 2 min ensuring, all the surfaces are cleaned; twice per day in the morning and evening before retiring to bed (16). 3. Use fluoride toothpaste to brush, spit the foam but, do not rinse it out (16). 4. Change your toothbrush every 3 months or when bristles flare out (16). 5. Advise your parent or guardian to buy toothpaste containing at least 1,450 ppm fluoride and to take you for a dental checkup at least once a year (16).

#### 2.3.2 Control group

The adolescents in the control group were left to continue with their routine daily oral care. Routine daily oral care includes all self-oral cleaning and seeking of oral treatment without any preventive intervention instructions from the modified BPOC protocol organizers. Participants found with emergence conditions during baseline data collection were referred to nearby dental clinics. At the end of the study, all participants received peer led oral health education based on five key messages (Box 1).

### 2.4 Study outcomes

The primary outcome measure of this study was the prevalence of dental caries. The study also measured knowledge on dental caries, the frequency of consuming sugary drinks and foods per day, the use of fluoridated toothpaste per day, and dental visits in the previous year. The primary and secondary outcomes were measured at baseline, at 18 and 24 months follow up intervals.

### 2.5 Data collection

#### 2.5.1 Validated self-administered questionnaire

A validated self-administered questionnaire was constructed using questions derived from standardized tools (17–19). The questionnaire assessed socio-demographic factors, which included age, sex, geographical location, parental education, and socioeconomic status. It also assessed the secondary objectives (knowledge and oral health behaviors) related to dental caries.

Participant's age was recorded as an absolute number ranging from 10–19, while sex was recoded as 1 = male and 2 = female. Geographical location was recorded as 1 = urban and 2 = rural based on Copperbelt province rural-urban delineation. Mother's and father's education were recorded according to Zambian education structure (18) as follows: 1 = no formal education, 2 = up to primary, 3 = secondary, 4 = tertiary (college or university) and thereafter dichotomized into [0 = up to primary (including the original scores 1–2) and 1 = Secondary or higher (including the original scores 3–4)]. Socio-economic status (SES) was derived from international wealth index (IWI) items (19) as elaborated in a previous publication (20) and recorded as 1 = high and 2 = low to middle.

Knowledge on dental caries was assessed using ten questions inquiring on causes, symptoms, and prevention of oral diseases, giving a minimum score of 0 and a maximum of 10. A cut off point of 7 correct responses was adopted for adequate knowledge; therefore, participants were categorized into those having inadequate knowledge [0 = (including the original scores 0–6)] and having adequate knowledge [1 = (including the original scores 7–10)]. Oral health related behaviors assessed were; frequency of consuming sugary drinks and foods per day in the past 30 days, frequency of use of fluoridated toothpaste per day in the past 30 days, and dental visits in the previous year. Frequencies of consuming sugary drinks and foods were scored as (1 = I didn't take, 2 = Occasionally per week, 3 = Once per day, 4 = Twice to four times per day, 5 = Five times or more per day) and thereafter dichotomized into [0 = less than 5 times per day (including the original scores 1–4)] and 1 = 5 times or more per day (including the original category 5). Frequency of use of fluoridated toothpaste per day was scored as; (1 = I didn't, 2 = I did but not every day, 3 = I did once a day, 4 = I did twice a day or more) and later dichotomized into [0 = less than twice per day (including original scores 1–3)] and 1 = twice or more per day (including original score 4). Visiting a dentist in the previous year was scored as (1 = I didn't attend, 2 = I attended once, 3 = I attended twice or more) and thereafter dichotomized into [0 = I didn't attend (including original score 1) and 1= I attended once or more (including original score 2–3)].

#### 2.5.2 Clinical examination

Dental caries on permanent dentition was examined by four trained and calibrated dentists. The training and calibration process was done in two stages, where in the first stage the principal investigator (PI) was trained by an experienced local dental public health epidemiologist (KN) in Tanzania, and in the second stage four data collection assistants were trained and calibrated in Zambia by the PI. Both training and calibration sessions involved discussion and agreement testing of the clinical presentation of CAST codes using clinical photographs and pre-selected school children with a full range of CAST codes. The trainer and trainees separately examined a set of 30 students in groups of 10 students at a time comparing CAST codes and then discussing any differences before taking the next set of 10 students. Calibration was considered sufficient when the trainer and the trainee agreement were at least 85%. The coefficients of reliability (Cohen's kappa) for CAST between and within examiners ranged from 0.80 to 0.85 and 0.80 to 0.90, respectively. Scored was done according to categories as described in the CAST manual by Frencken et al., (2015), as follows: 0 = sound, 1 = sealant, 2 = restoration, 3 = caries in enamel, 4 = caries in dentin without distinct cavitation (discolored dentin visible through enamel), 5 = caries in dentin with distinct cavitation, 6 = caries in pulp, 7 = abscess or fistula, 8 = lost due to caries, 9 = others (21, 22). The categories were thereafter grouped into five diagnostic thresholds of CAST as follows; {0 = CAST code 0–2 (healthy), 1 = CAST code 3–4 (pre-morbidity), 2 = CAST code 5 (morbidity), 3 = CAST code 6–7 (severe morbidity), and 4 = CAST code 8 (mortality). The prevalence of dental caries was assessed by dichotomizing CAST codes into two as “with caries” for CAST codes 3–7 and “without caries” for CAST codes 0–2, 8, and 9. A detailed description of the examination of dental caries is provided in the article reporting prevalence and factors associated with dental caries among this group (6).

### 2.6 Sample size and sampling

**Figure 2:**
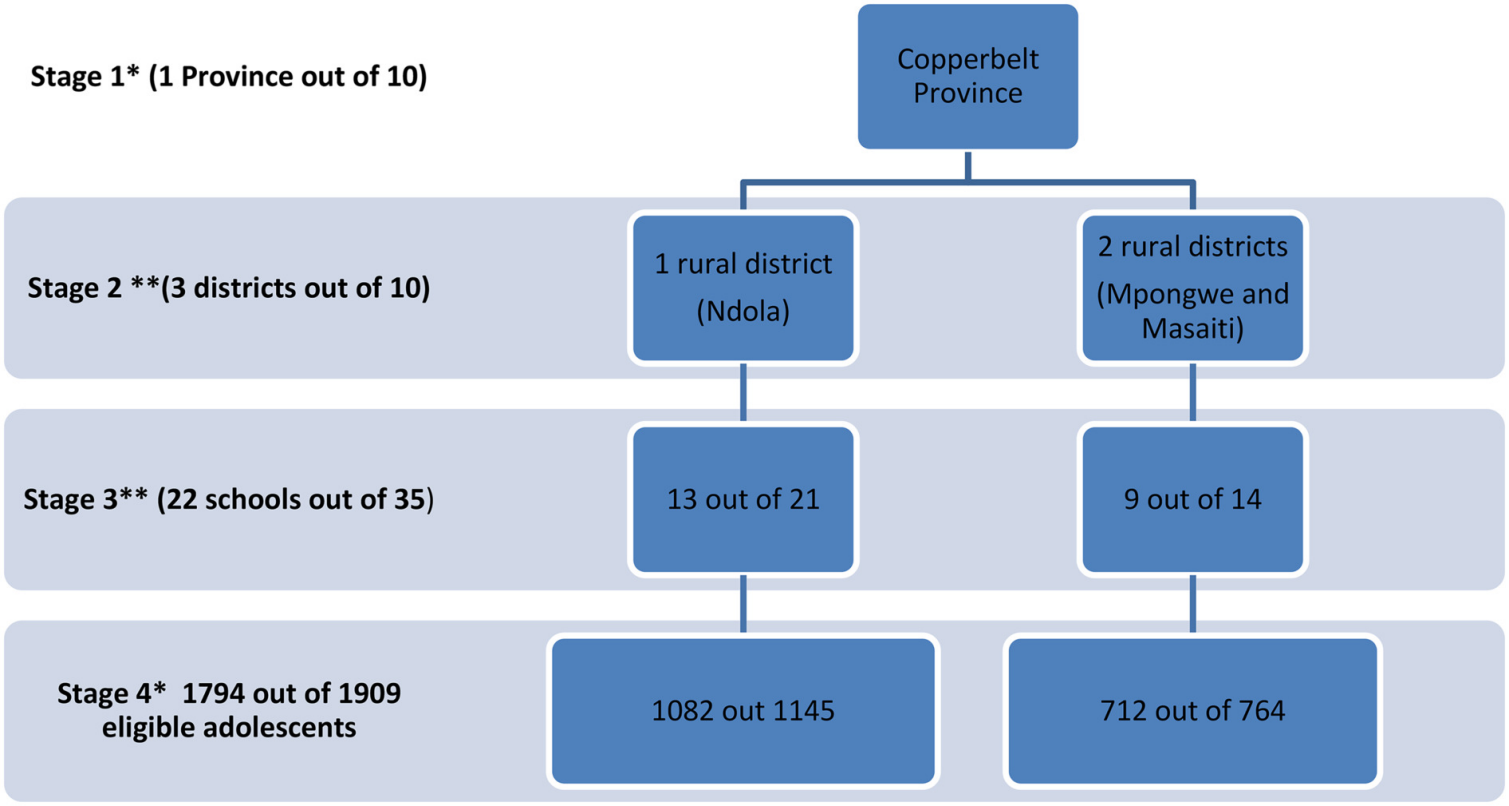
Sampling flow chart. Key *nonrandom sampling **random sampling.

A sample size of 1,760 participants was estimated by assuming: a 95% confidence level, 85% power, 5% margin of error, 20% expected mean change in dental caries, a cluster size of 80, and a mean DMFT of 1.34 found in a previous study involving 5–17 year-old pupils in Zambia (5). A multistage sampling method was employed to select adolescents in grades 8 and 9 attending 22 public secondary schools in three randomly selected districts of the Copperbelt province in Zambia. The Copperbelt Province was conveniently selected out of the ten provinces of Zambia at the first stage. The choice of Copperbelt Province was based on the intention to fit the study into existing community dentistry training at the university where the principal investigator is based. The second stage involved proportionate stratified random sampling of three districts (one urban and two rural) out of the ten districts of the Copperbelt province. In the third stage, a total of 22 out of 35 public secondary schools were randomly selected, guided by the number of schools in each district (21:35, =13 schools for Ndola district; 8:35, =5 schools for Masaiti district; and 6:35, =4 schools for Mpongwe district). In the fourth stage, all grade 8 and 9 adolescents enrolled in selected schools were eligible to participate in the study (Figure 2).

### 2.7 Randomization

The unit of randomization was secondary schools. The allocation ratio of 1:1 resulted in the inclusion of 11 schools in the intervention group (908 participants) and 11 in the control group (886 participants) at baseline (Figure 1). Stratified randomization was done in order to prevent an imbalance in the rural-urban characteristics of the clusters. Separate randomization was done for an urban district (Ndola) and rural districts (Masaiti and Mpongwe). Ndola (urban) clusters were first randomized in a block of 2 by taking the first cluster to intervention and the second to control. After completing the Ndola clusters, Masaiti and Mpongwe were randomized in the block size of 2. Sealed envelope (23) was used to create blocked randomization (Seed = 72084789304093, block size = 2, list length Ndola 13, actual list length Ndola = 14, list length for Masaiti and Mpongwe = 9, actual list length for Masaiti and Mpongwe = 10). The opaque envelopes were opened a day before intervention day. Randomization ended with 7 clusters in the intervention group and 6 in the control for the urban district (Ndola) and 4 clusters in the intervention and 5 in the control for rural districts (Masaiti and Mpongwe). The Principal Investigator (SA) generated the allocation sequence, four dentists enrolled participants, and an independent person assigned clusters into interventions.

### 2.8 Blinding

Single blinding was done whereby the examiners were not aware of group allocation during baseline and follow-up. Blinding of the participants was not feasible because the participants in the intervention group received peer delivered oral health education and treatment for dental caries.

### 2.9 Statistical methods

Data entry, cleaning, and analysis were done using IBM SPSS **(**version 26.0 IBM SPSS Statistics) and the significance level was set to *p* < 0.05. Analysis was based on an intention-to-treat approach, and generalized estimating equation (GEE) analysis was used to include all randomized participants. The baseline, first, and second follow up socio-demographics and clinical characteristics of the participants in the intervention and control groups are presented in frequency tables as percentages and frequencies. The patterns in the distribution of outcome variables across the three time points in intervention and control groups are also presented in a frequency table as percentages, frequencies and percentage point change at 18 and 24 months from baseline. To take into account correlated outcome observations within individuals due to repeated measurements, the likelihood of change in caries across study groups was analyzed using generalized estimating equations (GEE) with a binomial model, logit link function, and unstructured correlation structure (24). First, GEE analyses were used to analyze the overall intervention effect of the outcome variables, and secondly, interactions between the intervention/control group and time were explored to evaluate the intervention effects at follow up and whether the outcome variables showed different trajectories across time. Stratified analyses were performed for the outcome variables which showed significant interaction. Results of GEE are presented as adjusted odds ratios and their 95% confidence intervals.

## 3 Results

### 3.1 Participants flow diagram

A total of twenty-two schools were randomly allocated into intervention and control groups in a ratio of 1:1, resulting in 908 and 886 participants in the intervention and control groups, respectively as shown in Figure 1. The intervention group received modified BPOC from June to December 2021, while the control group did not receive any intervention and therefore continued with their routine oral care. There was no complete loss of follow up in any of the clusters at the two follow ups. A total of 64 participants in the intervention and 40 in the control group were lost to follow up after 18 months, while 117 participants in the intervention and 80 in the control group were lost to follow-up after 24 months, all of them due to being transferred from one school to another. A total of 1,794 participants (908 intervention, 886 control) were analyzed at baseline and after 18 and 24 months. The second follow up ended in March 2023.

### 3.2 Baseline characteristics

Baseline socio-demographics, oral health related knowledge and behaviors, and clinical indicators of the participants according to trial arm allocation are as shown in Table 1. Despite randomization, the chi-square test and variance analysis showed significant baseline differences between the intervention and control arms on the following socio-demographic characteristics; sex (*p* = 0.003), mothers' education (*p* = 0.023), fathers' education (*p* = 0.021), and socioeconomic status (*p* < 0.001). The intervention group had a higher proportion of females (57.4% vs. 50.5%), rural participants (56.8% vs. 33.2%), participants whose mothers (38.4% vs. 33.3%) and fathers (43.4% vs. 38.0%) had attained secondary school or higher education, and low to middle SES (52% vs. 35.3%) compared to controls. The results also showed baseline differences according to dental visits in the previous year (*p* = 0.004), and CAST clinical severity categories (*p* < 0.001). The intervention group had significantly higher percentages of those who did not attend dental visits (76.5% vs. 70.5%), and those with dental caries (51.3% vs. 41.3%) than the control group. Table 2 shows socio-demographics and clinical variables at baseline by response status at 18- and 24 months follow-up. The baseline socio-demographics (age, sex, residence, mothers' education, fathers' education), oral health related knowledge and behaviors, and clinical variables (caries) between those lost to follow up and those who remained in the study did not differ significantly (all *p*-values > 0.05).

**Table 1:** Socio-demographics, oral health knowledge, oral health behaviors, and clinical characteristics by intervention arms at baseline among a sample of Zambian adolescents (*n* = 1,794).

Variables	Categories	Trial arm allocation		*p*-value
		Intervention	Control	
		% (*n*)	% (*n*)	
Socio-demographics				
Age	11–14	54.4 (494)	57.6 (510)	0.178
	15–19	45.6 (414)	42.4 (376)	
Sex	Male	42.6 (387)	49.5 (439)	0.003
	Female	57.4 (521)	50.5 (447)	
Residence	Urban	43.2 (392)	66.8 (592)	<0.001
	Rural	56.8 (516)	33.2 (294)	
Mothers' education	Up to primary	61.6 (559)	66.7 (591)	0.023
	Secondary or higher	38.4 (349)	33.3 (295)	
Fathers' education	Up to primary	56.6 (514)	62.0 (549)	0.021
	Secondary or higher	43.4 (394)	38.0 (337)	
Socio-economic status	High	48.0 (436)	64.7 (573)	<0.001
	Low to middle	52.0 (472)	35.3 (313)	
Oral health knowledge	Inadequate knowledge	42.3 (384)	40.2 (356)	0.364
	Adequate knowledge	57.7 (524)	59.8 (530)	
Oral health related behaviors				
Frequency of intake of sugary drinks	less than 5 times per day	88.1 (800)	87.6 (776)	0.736
	5 times or more per day	11.9 (108)	12.4 (110)	
Frequency of intake of sugary foods	less than 5 times per day	87.9 (798)	88.7 (786)	0.586
	5 times or more per day	12.1 (110)	11.3 (100)	
Use of fluoridated toothpaste	less than 2 times per day	21.1 (192)	22.5 (199)	0.500
	2 times or more per day	78.9 (716)	77.5 (687)	
Dental visits in previous one year	Attended at least once	23.5 (213)	29.5 (261)	0.004
	Did not attend	76.5 (695)	70.5 (625)	
Clinical variables				
CAST clinical severity categories	Healthy (CAST0-2)	46.7 (424)	55.9 (495)	<0.001
	Pre-morbidity (CAST3)	25.9 (235)	23.7 (210)	
	Morbidity (CAST4-5)	17.8 (162)	11.4 (101)	
	Severe morbidity (CAST6-7)	7.5 (68)	6.2 (55)	
	Mortality (CAST8)	2.1 (19)	2.8 (25)	
Dental caries prevalence	Caries free	48.7 (442)	58.7 (520)	<0.001
	With caries	51.3 (466)	41.3 (366)	

**Table 2:** Socio-demographics and clinical variables at baseline by response status among a sample of Zambian adolescents (*n* = 1,794).

Variables	Categories	Follow ups					
		18 months			24 months		
		Lost	Followed	*p* value	Lost	Followed	*p* value
		% (*n*)	% (*n*)		% (*n*)	% (*n*)	
Socio-demographics							
Age	11–14	67.3 (70)	55.3 (934)	0.016	59.4 (117)	55.5 (887)	0.305
	15–19	32.7 (34)	44.7 (756)		40.6 (80)	44.5 (710)	
Sex	Male	48.1 (50)	45.9 (776)	0.668	44.7 (88)	46.2 (738)	0.682
	Female	51.9 (54)	54.1 (914)		55.3 (109)	53.8 (859)	
Residence	Urban	58.7 (61)	54.6 (923)	0.422	53.8 (106)	55.0 (878)	0.755
	Rural	41.3 (91)	45.4 (767)		46.2 (91)	45.0 (719)	
Mothers' education	Up to primary	65.4 (68)	64.0 (1,082)	0.779	62.9 (124)	64.2 (1,026)	0.719
	Secondary or higher	34.6 (36)	36.0 (608)		37.1 (73)	35.8 (571)	
Fathers' education	Up to primary	66.3 (69)	58.8 (994)	0.129	62.9 (124)	58.8 (939)	0.264
	Secondary or higher	33.7 (35)	41.2 (696)		37.1 (73)	41.2 (658)	
Socio-economic status	High	65.4 (68)	55.7 (941)	0.053	60.4 (119)	55.7 (890)	0.212
	Low to middle	34.6 (36)	44.3 (749)		39.6 (78)	44.3 (707)	
Oral health knowledge	Inadequate	38.5 (40)	41.4 (700)	0.552	41.6 (82)	41.2 (658)	0.910
	Adequate knowledge	61.5 (64)	58.6 (990)		58.4 (115)	58.8 (939)	
Oral health related behaviors							
Frequency of intake of sugary drinks	less than 5 times per day	88.5 (92)	87.8 (1,484)	0.844	88.3 (174)	87.8 (1,402)	0.828
	5 times or more per day	11.5 (12)	12.2 (206)		11.7 (23)	12.2 (195)	
Frequency of intake of sugary foods	less than 5 times per day	82.7 (86)	88.6 (1,498)	0.067	87.3 (172)	88.4 (1,412)	0.649
	5 times or more per day	17.3 (18)	11.4 (192)		12.7 (25)	11.6 (185)	
Use of fluoridated toothpaste	less than 2 times per day	16.3 (17)	22.1 (374)	0.166	19.8 (39)	22.0 (352)	0.472
	2 times or more per day	83.7 (87)	77.9 (1,316)		80.2 (158)	78.0 (1,245)	
Dental visits in previous one year	Attended at least once	26.0 (27)	26.4 (447)	0.871	26.9 (53)	26.4 (421)	0.871
	Did not attend	74.0 (77)	73.6 (1,243)		73.1 (144)	73.6 (1,176)	
Clinical variables							
CAST clinical severity categories	Healthy (CAST0-2)	51.9 (54)	51.2 (865)	0.853	52.3 (103)	51.1 (816)	0.750
	Pre-morbidity (CAST3)	25.0 (26)	24.8 (419)		27.4 (54)	24.5 (391)	
	Morbidity (CAST4-5)	11.5 (12)	14.9 (251)		12.7 (25)	14.9 (238)	
	Severe morbidity (CAST6-7)	8.7 (9)	6.7 (114)		5.6 (11)	7.0 (112)	
	Mortality (CAST8)	2.9 (3)	2.4 (41)		2.0 (4)	2.5 (40)	
Dental caries prevalence	Caries free	54.8 (57)	53.6 (905)	0.803	54.3 (107)	53.5 (855)	0.837
	With caries	45.2 (47)	46.4 (785)		45.7 (90)	46.5 (742)	

#### 3.2.1 Descriptive statistics of outcome variables according to trial intervention and across time

Table 3 shows the trends in the distributions of participants according to knowledge, oral health- related behaviors, and dental caries by intervention group and across time. The results show a statistically significant difference between groups after 18 months (*p* = 0.014) and 24 months (*p* = 0.002). The percentage change in the proportion of participants with adequate knowledge from baseline to 18-and-24 months follow up intervals was significant in the intervention group at 18 months [9.6% (5.1%, 14.1%), *p* < 0.001] and 24 months [11.5% (5.1%, 14.1%), *p* < 0.001]}. The use of fluoridated toothpaste in the intervention group increased by 4.5% (0.8%, 8.1%), *p* = 0.016 and 4.4% (0.6%, 8.1%), *p* = 0.021 at 18 and 24 months respectively. The frequency of dental visits was lower in the intervention than control group (23.5% vs. 29.5%, *p* < 0.001) at baseline but changed to no difference at 18 months (*p* = 0.733) and 24 months (*p* = 0.688). Dental visit in the intervention group increased by 5.6% [5.6% (1.5%, 9.7%) *p* = 0.008] and 5.4% [5.4% (1.2%, 9.6%) *p* = 0.011] at 18 and 24 months, respectively. The prevalence of dental caries was significantly higher in the intervention group than in the control group at baseline, 18 months and 24 months (*p* < 0.001). The prevalence of dental caries from baseline to 18 months' follow-up and from baseline to 24 months' follow-up did not change significantly either in the intervention- nor in the control groups. The percentage change, 95% confidence interval, and *p* values for the intervention at 18 months and 24 months were [−2.5% (−2.2%, 7.2%) *p* = 0.296], and [−2.6% (−2.2%, 7.3%) *p* = 0.285] respectively. The corresponding change estimates in the control group were [ −0.9 (−3.7, 5.5), *p* = 0.703] at 18 months and [−2.3 (−2.3, 6.9), *p* = 0.335] at 24 months.

**Table 3:** Distributions of participants according to knowledge, oral health related behaviors, and dental caries by trial arm across time among a sample of Zambian adolescents (*n* = 1,794).

Outcome variable	Percentage and number across time			Percentage point change from baseline (95% CI) and *p* value	
	Baseline	18 months	24 months	at 18 months	at 24 months
	% (*n*)	% (*n*)	% (*n*)	% Δ (95% CI), *p* value	% Δ (95% CI), *p* value
Knowledge (adequate)					
Intervention	57.7 (524)	67.3 (568)*	69.2 (547)*	9.6 (5.1, 14.1), *p* < 0.001	11.5 (6.9, 15.9), *p* < 0.001
Control	59.8 (530)	61.6 (521)	61.9 (499)	1.8 (−2.8, 6.4), *p* = 0.443	2.1 (−2.6, 6.7), *p* = 0.377
Oral health behaviors					
Sugary drinks per day (less than 5 times)					
Intervention	88.1 (800)	87.6 (739)	87.2 (689)	−0.5 (−2.5, 3.6), *p* = 0.749	−0.9 (−2.2, 4.1), *p* = 0.573
Control	87.6 (776)	86.9 (735)	86.6 (698)	−0.7 (−2.4, 3.9), *p* = 0.662	1.0 (−2.2, 4.2), *p* = 0.539
Sugary foods per day (less than 5 times)					
Intervention	87.9 (798)	87.7 (740)	87.3 (690)	−0.2 (−2.9, 3.3), *p* = 0.898	−0.6 (−2.6, 3.8), *p* = 0.708
Control	88.7 (786)	88.8 (751)	88.3 (712)	0.1 (−2.9, 3.1), *p* = 0.947	−0.4 (−2.6, 3.5), *p* = 0.797
Fluoridated toothpaste per day (2 times/more)					
Intervention	78.9 (716)	83.4 (704)*	83.3 (658)*	4.5 (0.8, 8.1), *p* = 0.016)	4.4 (0.6, 8.1), *p* = 0.021
Control	77.5 (687)	77.1 (652)	77.3 (623)	−0.4 (−3.5, 4.4), *p* = 0.843	−0.2 (−3.8, 4.2), *p* = 0.922
Dental visits in previous one year (attended)					
Intervention	23.5 (213)*	29.1 (246)	28.9 (228)	5.6 (1.5, 9.7), *p* = 0.008	5.4 (1.2, 9.6), *p* = 0.011
Control	29.5 (261)	29.9 (253)	29.8 (240)	0.4 (−3.9, 4.7), *p* = 0.856	0.3 (−4.1, 4.7), *p* = 0.893
Dental caries (with caries)					
Intervention	51.3 (466)**	48.8 (412)*	48.7 (385)*	−2.5 (−2.2, 7.2), *p* = 0.296	−2.6 (−2.2, 7.3), *p* = 0.285
Control	41.3 (366)	40.4 (342)	39.0 (314)	−0.9 (−3.7, 5.5), *p* = 0.703	−2.3 (−2.3, 6.9), *p* = 0.335

Percentage and numbers given are for only one category of the outcome variable as indicated in bracket in front of the outcome variable.

* *p* value < 0.05.

** *p* < 0.001, Δ = percentage.

### 3.3 The outcome of modified BPOC at 18 and 24 months (generalized estimating equations)

Generalized estimating equation models were used to assess the outcome of the modified BPOC intervention on knowledge, oral health behaviors, and dental caries prevalence at 18 months (Table 4) and 24 months follow up (Table 5). At 18 months (Table 4), a significant interaction effect between time and group on adequate knowledge occurred across time after adjustment for the main effects of group and time (modified BPOC group × time interaction) {OR (95% CI) = 1.3 (1.1, 1.5), *p* < 0.001}. The model for use of fluoridated toothpaste twice or more per day also showed significant interaction between modified BPOC group and time [OR (95% CI) = 1.6 (1.3, 2.1), *p* < 0.001)]. The dental caries interaction model at 18 months was also significant [OR (95% CI) = 0.7 (0.6, 0.8), *p* < 0.001)]. Table 5 shows the results from GEE models regarding the outcome of the modified BPOC intervention at 24 months follow-up. The intervention group (modified BPOC group × time interaction) for adequate knowledge model was significant at 24 months [OR (95% CI) = 1.6 (1.3, 2.0)], indicating group difference in knowledge across time from baseline to 24 months follow-up. Among the oral health related behaviors, use of fluoridated toothpaste twice or more per day showed significant interaction between the modified BPOC group and time [OR (95% CI) = 1.6 (1.3, 2.1), *p* < 0.001)] at 24 months. The dental caries interaction model at 24 months was also significant [OR (95% CI) = 0.7 (0.6, 0.8), *p* < 0.001)].

**Table 4:** Generalized estimating equation analyses of the outcome variables (knowledge, behaviors, and dental caries) for the study sample of Zambian adolescents from baseline to 18 months (*n* = 1,794).

Outcome variable	Parameters	Sig.	OR (95% CI)
Adequate Knowledge			
Main effects	Time	<0.001	1.3 (1.2, 1.4)
	mBPOC group	0.154	1.1 (1.0, 1.4)
Interaction effects	mBPOC group × time	0.022	1.3 (1.1, 1.5)
Behaviors			
Sugary drinks per day (less than 5 times)			
Main effects	Time	0.207	0.9 (0.8, 1.0)
	mBPOC group	0.790	1.0 (0.7, 1.3)
Interaction effects	mBPOC group × time	0.506	0.9 (0.7, 1.2)
Sugary foods per day (less than 5 time)			
Main effects	Time	0.873	1.0 (0.8, 1.1)
	mBPOC group	0.515	0.9 (0.7, 1.2)
Interaction effects	mBPOC group × time	0.505	0.9 (0.7, 1.2)
Fluoridated toothpaste per day (2 times/more)			
Main effects	Time	0.001	1.1 (1.1, 1.3)
	mBPOC group	0.004	1.4 (1.1, 1.7)
Interaction effects	mBPOC group × time	<0.001	1.6 (1.3, 2.1)
Dental visits in previous one year (attended)			
Main effects	Time	<0.001	1.2 (1.1, 1.3)
	mBPOC group	0.470	0.9 (0.8, 1.1)
Interaction effects	mBPOC group × time	0.422	1.1 (0.9, 1.4)
Dental caries (with caries)			
Main effects	Time	<0.001	0.9 (0.8, 0.9)
	mBPOC group	<0.001	1.4 (1.2, 1.7)
Interaction effects	mBPOC group × time	<0.001	0.7 (0.6, 0.8)

OR, odds ratio; CI, confidence intervals; Sig., significancy; mBPOC, modified basic package of oral care. results provided are based on intervention group and at 18 months with reference to control group and baseline data.

**Table 5:** Generalized estimating equation analyses of the outcome variables (knowledge, behaviors, and dental caries) for the study sample of Zambian adolescents from baseline to 24 months (*n* = 1,794).

Outcome variable	Parameters	Sig.	OR (95% CI)
Adequate Knowledge			
Main effects	Time	<0.001	1.3 (1.2, 1.4)
	mBPOC group	0.162	1.1 (0.9, 1.4)
Interaction effects	mBPOC group × time	<0.001	1.6 (1.3, 2.0)
Behaviors			
Sugary drinks per day (less than 5 times)			
Main effects	Time	0.089	0.9 (0.8, 1.0)
	mBPOC group	0.764	0.9 (0.7, 1.3)
Interaction effects	mBPOC group × time	0.394	0.9 (0.7, 1.2)
Sugary foods per day (less than 5 time)			
Main effects	Time	0.180	0.9 (0.8, 1.0)
	mBPOC group	0.520	0.9 (0.7, 1.2)
Interaction effects	mBPOC group × time	0.317	0.8 (0.6, 1.2)
Fluoridated toothpaste per day (2 times/more)			
Main effects	Time	<0.001	1.2 (1.1, 1.3)
	mBPOC group	0.011	1.3 (1.1, 1.7)
Interaction effects	mBPOC group × time	<0.001	1.6 (1.3, 2.1)
Dental visits in previous one year (attended)			
Main effects	Time	<0.001	1.2 (1.1, 1.3)
	mBPOC group	0.371	0.9 (0.7, 1.1)
Interaction effects	mBPOC group × time	0.596	1.1 (0.9, 1.3)
Dental caries (with caries)			
Main effects	Time	<0.001	0.9 (0.8, 0.9)
	mBPOC group	<0.001	1.5 (1.2, 1.8)
Interaction effects	mBPOC group × time	<0.001	0.7 (0.6, 0.8)

OR, odds ratio; CI, confidence intervals; Sig., significancy; mBPOC, modified basic package of oral care. results provided are based on intervention group and at 24 months with reference to control group and baseline data.

### 3.4 Stratified analysis

Stratified analyses of the outcome variables with significant interactions between BPOC group and time at 18 and 24 months are as shown in Table 6. The findings show that the likelihoods of having adequate knowledge at 18- and 24-months follow ups compared to baseline were stronger in the intervention group than in the control group. The odds ratio, 95% confidence intervals, and *p* values for adequate knowledge at 18 months were [OR (95% CI) = 1.5 (1.4, 1.7), *p* < 0.001)] for the intervention and [OR (95% CI) = 1.1 (1.0, 1.2), *p* = 0.020)] for the control group compared to baseline values. The odds ratios, 95% confidence intervals, and *p* values for the intervention and control groups at 24 months compared to baseline values were [OR (95% CI) = 1.6 (1.5, 1.9), *p* < 0.001)] and [OR (95% CI) = 1.1 (1.0, 1.2), *p* = 0.020)] respectively. The likelihood of using fluoridated toothpaste two times or more per day at 18 and 24 months of follow up compared to baseline was stronger in the intervention group than in the control group. The use of fluoridated toothpaste increased by about 1.4 times at both 18 months [OR (95% CI) = 1.4 (1.2, 1.6), *p* < 0.001)] and 24 months [OR (95% CI) = 1.4 (1.2, 1.5), *p* < 0.001)] in the intervention group but did not change significantly for control group. The likelihood of having dental caries at 18 and 24 months follow up compared to baseline decreased more in the intervention than in the control group. The odds of dental caries in the intervention group decreased by 20% at both 18 months [OR (95% CI) = 0.8 (0.8, 0.9), *p* = 0.013)] and 24 months [OR (95% CI) = 0.8 (0.8, 0.9), *p* = 0.021)] compared to 10% decrease in the control group at 18 months [OR (95% CI) = 0.9 (0.8, 0.9), *p* = 0.002)] and 24 months [OR (95% CI) = 0.9 (0.8, 0.9), *p* < 0.001)].

**Table 6:** Modified BPOC outcome variables regressed on time (18- and 24-months follow up vs. baseline) separately in the intervention (*n*_I_ = 908) and control (*n*_c_ = 886) groups among the study sample of Zambian adolescents (*n* = 1,794).

Follow up	Outcomes	Categories	Groups			
			Intervention		Control	
			Sig.	OR (95% CI)	Sig.	OR (95% CI)
At 18 months	Adequate Knowledge	18 months	<0.001	1.5 (1.4, 1.7)	0.020	1.1 (1.0, 1.2)
		Baseline		1		1
	Fluoridated toothpaste per day (2 times/more)	18 months	<0.001	1.4 (1.2, 1.6)	0.934	1.0 (0.9, 1.1)
		Baseline		1		1
	Dental caries (with caries)	18 months	0.013	0.8 (0.8, 0.9)	0.002	0.9 (0.8, 0.9)
		Baseline		1		1
At 24 months	Adequate Knowledge	24 months	<0.001	1.6 (1.5, 1.9)	0.020	1.1 (1.0, 1.2)
		Baseline		1		1
	Fluoridated toothpaste per day (2 times/more)	24 months	<0.001	1.4 (1.2, 1.5)	0.935	1.0 (0.9, 1.1)
		Baseline		1		1
	Dental caries (with caries)	24 months	0.021	0.8 (0.8, 0.9)	<0.001	0.9 (0.8, 0.9)
		Baseline		1		1

## 4 Discussion

This study investigated the impact of a modified basic package of oral care intervention on the prevalence of dental caries as well as knowledge and oral health behaviors related to dental caries among adolescents in Copperbelt Province, Zambia. The null hypotheses for the primary objective (dental caries prevalence) and some of the secondary objectives (knowledge, use of fluoridated toothpastes) were rejected. However, those for consuming sugary drinks and foods less than five times per day and visiting a dentist at least once in the past year were accepted.

The major findings of the current study indicate that at follow ups after cessation of the modified BPOC intervention, knowledge on dental caries and frequency of using fluoridated toothpastes among participants in the modified BPOC group improved over time. The frequency of consumption of sugary drinks and foods did not significantly change over time for either the intervention or control group. Over time, the prevalence of dental caries decreased in both groups; however, the intervention group had a twofold decrease in dental caries compared to the control group. The findings of this study set a platform to inform national and international oral health stakeholders such as the governments, health ministries, oral health researchers, and toothpaste manufacturers that the basic package of oral care (BPOC) is feasible and effective in managing o dental caries at various stages of caries process. Early detection of caries during childhood and adolescence particularly using the caries assessment and treatment spectrum (CAST) and its management using BPOC intervention, should be adopted by the Zambian government and Africa at large for the benefit of our patients.

Improvement in knowledge among the participants in the modified BPOC group was likely attributed to repetitive oral health messages delivered by peers. Comparable improvements in knowledge have been reported in previous studies that used peer-led oral health education (25–27).

Surprisingly, the observed positive change in knowledge in the modified BPOC group in this study did not lead to favorable behavior change over time. The lack of favorable change in the consumption of sugary foods and drinks despite improvement of knowledge could be attributed to immaturity in the brain pathways involved in decision-making, making adolescents sensitive to emotional and social influences and therefore having difficulty controlling their lifestyle choices (28). Adolescents are also dependents thus, their eating behaviors are not only influenced by their knowledge but also by what foods are available and accessible to them, which ultimately influences what they consume (29). This finding highlights the impact of other determinants of behavior such as the environment, cultural and social norms, and social support from friends (peers) and parents that influence behavior change, which were not evaluated or controlled in this cluster randomized trial (30). In contrast to the current findings, other authors (25–27) have reported positive changes in both knowledge and behavior following peer oral health education.

Participants in the modified BPOC group used fluoridated toothpaste more frequently than controls in this study. This could be attributed not only to increased knowledge about dental caries, which was given through peer-led OHE, but also to motivation to continue using oral cleaning products after receiving free toothpaste during the six-month intervention period. The importance of knowledge, particularly the benefits of fluoride use in caries prevention, constitutes one of the major factors influencing adolescents' use of fluoridated toothpaste (31). The more significant improvement in the intervention group than the control group, regarding usage of fluoridated toothpaste is comparable with the findings of a previous trial in which free toothpaste and toothbrushes were distributed along with messages and instructions on daily brushing (32).

The treatment components of the modified BPOC intervention, which included extraction of teeth and atraumatic restorative treatment, could explain the two-fold decrease in the prevalence of dental caries among participants in the modified BPOC compared to the control group. Frequent use of fluoridated toothpaste in the modified BPOC group compared to controls may also have contributed to a higher improvement in caries status in the modified BPOC group. Comparison with studies on the effectiveness of the BPOC intervention in caries prevention among adolescents was not possible due to the scarcity of similar studies. However, the findings are consistent with those of a study that investigated the effectiveness of oral urgent treatment, one of the four components of BPOC, in lowering the dental caries burden in Filipino children (33).

The findings of this study need to be interpreted within the strengths and limitations of a cluster randomized trial. The study involved a large sample size from a total of twenty-two clusters, which exceeded the recommended minimum number of clusters for parallel randomized cluster trials of four per arm in order to obtain a *p*-value less than 0.05 under a randomization-based test (34). The findings can probably be generalized to adolescents attending public secondary schools in Copperbelt Province, Zambia, due to random selection and a large sample size. The design employed in this study allowed for the prevention of contamination of the intervention, which could lead to false rejection of an effective intervention due to a reduction of point estimates of its effectiveness (35). Data collection assistants were blinded in order to avoid bias in their assessment of dental caries status, which could ultimately affect the effectiveness of an intervention (36). However, it was not possible to blind the participants due to the nature of the modified BPOC intervention, which involved peer oral education, the provision of fluoridated toothpastes, and extraction and restoration of teeth. It is also important to note that despite randomization, there were baseline differences in dental caries and dental visits as well as in some socio-demographics between the intervention and control group, which could affect the treatment effects of the modified BPOC intervention. The use of a repeated measure (GEE) and reporting the interaction between the treatment variable and time in the models, reduces the bias of baseline differences (37). The short follow-up period and age group did not allow for a proper evaluation of the preventive effect of affordable fluoride and atraumatic restorative treatment.

## 5 Conclusions

The modified basic package of oral care was effective in reducing the prevalence of dental caries, improving knowledge on dental caries, and increasing the frequency of using fluoridated toothpaste among Zambian adolescents. The intervention did not have any effect on adolescents' frequency of consuming sugary drinks and foods. Further studies with longer follow up duration need to be conducted in order to fully assess the primary preventive effect of atraumatic restorative treatment (ART) and affordable fluoride toothpaste (AFT) components of BPOC. Policy makers need to consider adopting and incorporating BPOC in primary health care packages as an implementation of universal oral health care for children in Zambia.

## Data Availability

The datasets presented in this study can be found in online repositories. The names of the repository/repositories and accession number(s) can be found in the article/https://doi.org/10.6084/m9.figshare.26299045.v3/Supplementary Material.
