# Supplementary material for: The effect of the modified basic package of oral care on adolescent dental caries status in Zambia; a cluster randomized trial
Source: Front Oral Health. 2025 May 7;6:1542337. doi: 10.3389/froh.2025.1542337 (PMC12092211; doi:10.3389/froh.2025.1542337)
Supplement: Supplementary file 1 [file Datasheet1.pdf]

MUHIMBILI UNIVERSITY OF HEALTH AND ALLIED SCIENCES

SCHOOL OF DENTISTRY

DEPARTMENT OF ORTHODONTICS PAEDODONTICS & COMMUNITY DENTISTRY

PhD RESEARCH PROPOSAL

EFFECTIVENESS OF MODIFIED BASIC PACKAGE OF ORAL CARE ON ORAL HEALTH STATUS AND RELATED QUALITY OF LIFE AMONG ZAMBIAN ADOLESCENTS-: A FIELD TRIAL

Candidate Name: Dr Severine Nyerembe Anthony

Registration Number: HD/MUH/T.1000P/2020

Supervisors

Muhimbili University of Health and Allied Sciences

1. Prof Febronia Kokulengya Kahabuka
2. Dr Hawa Sharrif Mbawalla

Copperbelt University

1. Prof Seter Siziya

University of Bergen

1. Prof Anne Nordrehaug Åstrøm

Date: 10th August 2020

Table of Contents

Abbreviations iii

Definition of key terms iv

Summary vi

1.0 Introduction 1

1.1 Background 1

1.2. Problem statement 4

1.3. Conceptual framework 5

1.4. Rationale 6

1.5. Research hypothesis and Questions 8

2.0. Objectives 9

2.1. Broad Objective 9

2.2. Specific Objectives 9

3.0. Literature review 10

4.0. Materials and Methods 17

4.1. Study design 17

4.2. Study area 17

4.3. Study population 17

4.4. Sample size 18

4.5. Sampling 19

4.6. The trial intervention 19

4.7. Randomization 21

4.8. Inclusion and exclusion 21

4.8.1. Cluster level 21

4.8.2. Individual level 21

4.9. Data collection procedures 22

4.9.1. Data collection tools and indices 22

4.9.2. Blinding 23

4.10. Study outcomes 23

4.11. Study Variables 23
4.11.1. Independent variables 23
4.11.2. Dependent variables 24
Investigation tools validity and reliability 27
4.12. Data entry cleaning and analysis 28
4.13. Ethical considerations 28
4.14. Study limitation and mitigation 29
4.15. Dissemination of findings 29
5.0. Budget 31
6.0 Activity Plan 34
7.0 References 36
8.0 Appendices 49

Abbreviations

AFT	-	Affordable Fluoride Toothpaste
ART	-	Atraumatic Restorative Treatment
BPOC	-	Basic Package of Oral Care
CAST	-	Caries Assessment Spectrum and Treatment
DMFT	-	Decayed Missing Filled Teeth
DEBS	-	District Education Board Secretaries
ICC	-	Intra-Cluster Correlation Coefficient
LMICs	-	Low and middle-income countries
MUHAS	-	Muhimbili University of Health and Allied Sciences
NCDs	-	Non-Communicable Diseases
OHE	-	Oral Health Education
OHRQoL	-	Oral Health Related Quality of Life
OIDP	-	Oral Impacts on Daily performance
OUT	-	Oral Urgent Treatment
SB-FMR	-	School Based Fluoride Mouth Rinse
SES	-	Socioeconomic status
SSA	-	Sub Saharan Africa
STB	-	Supervised Tooth Brushing
TDRC	-	Tropical Diseases Research Control Institute
WHO	-	World Health Organization

Definition of key terms

Adolescent

World Health Organization (WHO) defines an adolescent as any person between ages 10 and 19, although other sources incorporate a span of 9 to 26 years (Curtis, A.C., 2015). WHO definition will be adopted in this study.

Basic Package of Oral Care (BPOC):

Is an intervention comprising of oral urgent treatment (OUT); atraumatic restorative treatment (ART) and affordable fluoride toothpaste (AFT) designed by WHO to improve oral health in low and middle-income countries (Batra *et al.*, 2014).

Dental caries

Is a biofilm-mediated, dynamic multi-factorial disease that results in the phasic demineralization and remineralization of dental hard tissues (Pitts *et al.*, 2017). In this study diagnosis of dental caries will include any visible change of enamel color or **translucency** to extensive distinct cavity with visible dentin or pulp exposure.

Dental plaque

Is a sticky colorless or pale yellow to brown biofilm or mass of bacteria that grows on tooth surface (Murakami *et al.*, 2018).

Gingivitis

Is a site-specific inflammatory condition initiated by dental biofilm accumulation and characterized by gingival redness and edema and the absence of periodontal attachment loss (Trombelli *et al.*, 2018).

Oral health

Is the ability to speak, smile, taste, chew, swallow and convey a range of emotions through facial expressions with confidence and without pain, discomfort and disease of the craniofacial complex (Lee *et al.*, 2017).

Oral health related quality of life

Is defined as a multidimensional construct that reflects (among other things) people's comfort when eating, sleeping and engaging in social interaction; their self esteem; and their satisfaction with respect to their oral health (Baiju *et al.*, 2017).

Phase III trials

Research which aims to provide a definitive assessment of the effectiveness of the intervention against the primary outcome(s) of interest (Smith et al., 2015).

Socio-demographic inequalities in health

Is any measurable aspect of health that varies across individuals or according to socially relevant groupings such as place of residence, race/ethnicity, occupation, gender, religion, education, socioeconomic status (SES), and social capital or resources (Arcaya et al., 2015).

Standard of self-care

encompass oral health care which a participant used to perform before enrollment or will perform during the study such as brushing of teeth, use of fluoridated tooth paste or attending for dental checkup or treatment" without being influenced or monitored by the study administrators.

Summary

Background: Global trends of diseases show an increase of oral diseases in most low and middle- income countries due to increased availability of sugars, inadequate fluoride products and lack of sustainable preventive strategies. Most oral diseases are behavioral related and behaviors acquired during adolescence are likely to be passed to adulthood. Adolescent's oral health behaviors are shaped by family socioeconomic status and therefore socio gradient in oral diseases may be more reflected in this group. Despite increasing oral diseases and Word Health Organization (WHO) recommendations of using BPOC as feasible and cost-effective preventive and curative approach of oral diseases, the strategy has not been implemented in Sub-Saharan Africa region.

Aim: To evaluate the effectiveness of applying the modified WHO basic package of oral care (BPOC) in improving oral health-knowledge and behaviors, oral health and related quality of life among adolescents in Copperbelt, Zambia.

Materials and methods: A cluster randomized controlled field trial with a total sample size of 1760 grade eight secondary school adolescents will be done in Copperbelt province, Zambia. Adolescents in the trial arm will receive a modified WHO-BPOC and the control will maintain their standard of care, both groups will be re-assessed at follow-ups. Data collection on adolescents' demographics, oral health-related knowledge and behavior, impacts of oral disease on daily performance and parental socio-demographics will be done using a self-administered structured questionnaire. Data on dentition status, plaque, and gingival bleeding will be assessed using CAST, Silness, and Loe (1967) plaque and gingival bleeding indices, respectively. Data entry, cleaning, and analysis will be done using IBM SPSS for windows (version 23), and summarized as frequency distribution and cross-tabulations. Adjusted binary logistic regression and generalized estimating equation will be performed to examine the differences between the groups at baseline and follow up in oral health-related behaviors, plaque, gingival bleeding, OHRQoL and dental caries status for various socio-demographics characteristics. The statistical significance difference shall be assumed when **p** ≤ 0.05.

Budget of the study: A total budget of TSh 34,341,450.00 is required to facilitate proposal writing, ethical clearance, purchase of dental materials and supplies, thesis writing and other logistics.

1.0 Introduction

1.1 Background

Oral health is an essential part of general health and wellbeing of individuals and societies as it affects general health and quality of life (Lee *et al.*, 2017). Adolescents' oral health depends on oral health-related behaviors (OHRB) such as frequency of tooth brushing, use of fluoridated toothpaste, regular preventive dental checkups, and frequency of consumption of sugary snacks and drinks. Oral health-related behaviors acquired during adolescence are likely to be passed on to adulthood and affect adulthood oral health signifying the importance of this age group (McDade *et al.*, 2011). Socio-economic status of the family influences OHRB as it affects recognition of the importance of oral health, access and affordability to oral health care products such as toothbrushes and fluoridated toothpastes and determines access to preventive and curative services (Jepsen *et al.*, 2017).

Socio-demographic gradient in health is any measurable aspect of oral health that varies across individuals or according to socially relevant groupings such as place of residence, race/ethnicity, occupation, gender, religion, education, socioeconomic status (SES), and social capital or resources (Arcaya et al., 2015). With respect to oral health, oral diseases disproportionately affect the poor and socially-disadvantaged members of societies, even in countries with the most established and universal welfare policies (Bergqvist et al,. 2013). Socio-economically deprived individuals tend to have worse clinical oral health and lower Oral Health Related Quality of Life (OHRQoL).

Dental caries and gingivitis are the most common behavioral related oral diseases which affect 40 to 90% of adolescents in LMICs (Benzian *et al.*, 2017). The mean DMFT among 12 years adolescents in African countries currently ranges from 0.3 to 4.9 whereas more than 90% of caries remain untreated (Abid *et al.*, 2015). Severe periodontal problems are uncommon among adolescents, however, gingivitis a mild form of periodontal diseases affects more than 50% of the adolescents before puberty (Abid *et al.*, 2018). In Sub-Saharan Africa, a higher prevalence of dental caries is reported more among girls than boys, individuals of higher than low socioeconomic background and urban than rural residents (Okullo et., 2004; Artemisa *et*

al., 2010; Mashoto *et al.*, 2010). Poor oral hygiene and periodontal conditions are more reported among boys than girls, individuals with lower than higher socioeconomic status, those living in rural than urban areas and older than younger adolescents (Varenne et al., 2006, Kolawole et al., 2011).

Oral Health Related Quality of Life (OHRQoL) is a concept that reflects people's comfort when eating, sleeping and engaging in social interaction; their self esteem; and their satisfaction with respect to oral health (Baiju *et al.*, 2017). Oral diseases negatively impact on the oral health-related quality of life of children and adolescents (Leal *et al.*, 2012; Peres *et al.*, 2013). Poor oral health during adolescence affects adolescent's food intake, self-esteem, disturb sleep, due to pain and interfere with participation in school activities leading to poor school performance (Ribeiro *et al.*, 2018). Impacts such as pain and perception of appearance are more severe in adolescents than adults as they not only affect their quality of life but also psychological development and social interactions (Peres *et al.*, 2013). A Systematic review of factors influencing oral health-related quality of life in children and adolescents in Africa report Child –OIDP as the most commonly used and validated measure of OHRQoL (Malele-Kolisa *et al.*, 2019). Recent studies in Sub- Saharan Africa (SSA) which used Child-OIDP reported poor oral health- related quality of life in terms of oral impact on daily performance ranging from 35.6 to 54.6% among adolescents. Economically disadvantaged adolescents report significantly poorer quality of life than the socioeconomically advantaged (Mashoto *et al.*, 2010; Mbawalla, *et al.*, 2010; Nurelhuda *et al.*, 2010).

Basic Package of Oral Health Care (BPOC) is an intervention recommended by the World Health Organization (WHO) as an affordable and effective preventive and curative modality of oral diseases in developing countries (Batra *et al.*, 2014). The intervention comprises of three components; Oral Urgent Treatment (OUT), Atraumatic Restorative Treatment (ART) and Affordable Fluoride Toothpastes (AFT). Safety and how well the components of the intervention works have been proved in previous studies (Kikwilu et al., 2009; Mashoto *et al.*, 2010), however effectiveness and implementation of the strategy as a package has not been tested in most African countries including Zambia. Modification of the conventional BPOC in this study entail inclusion of oral health education and provision of fluoridated tooth pastes to

encourage use of the oral care products in future. The conventional BPOC make fluoride tooth pastes available and affordable through regulating policies such as reduction or eliminating tax to the products aiming at reducing their market price. This is a level three trial that aims at assessing the effectiveness of the modified BPOC in improving oral health-related behaviors, oral health and related quality of life.

1.2. Problem statement

Dental caries and periodontal diseases affect 40 to 90% of adolescents in LMICs. Most of these diseases remain untreated, thus impacting negatively the adolescents' general health, well-being and quality of life, school performance, and social interactions. Furthermore, in SSA region the diseases accumulate in socio-demographically disadvantaged adolescents in situations where the available oral health systems and services are inadequate to cater for preventive and curative care. The cost of treating dental caries with the curative approach alone exceeds fiscal capabilities of most LMICs including Zambia. Despite evidence of increasing oral diseases and WHO recommendations of using BPOC as a cost-effective oral disease preventive and curative approach the strategy has not been tested and implemented in the Sub-Saharan Africa region, Zambia inclusive. Therefore, this study aims at assessing the effectiveness of the modified BPOC in improving oral health knowledge and related behaviors, oral health and related quality of life among Zambian Adolescents.

1.3. Conceptual framework

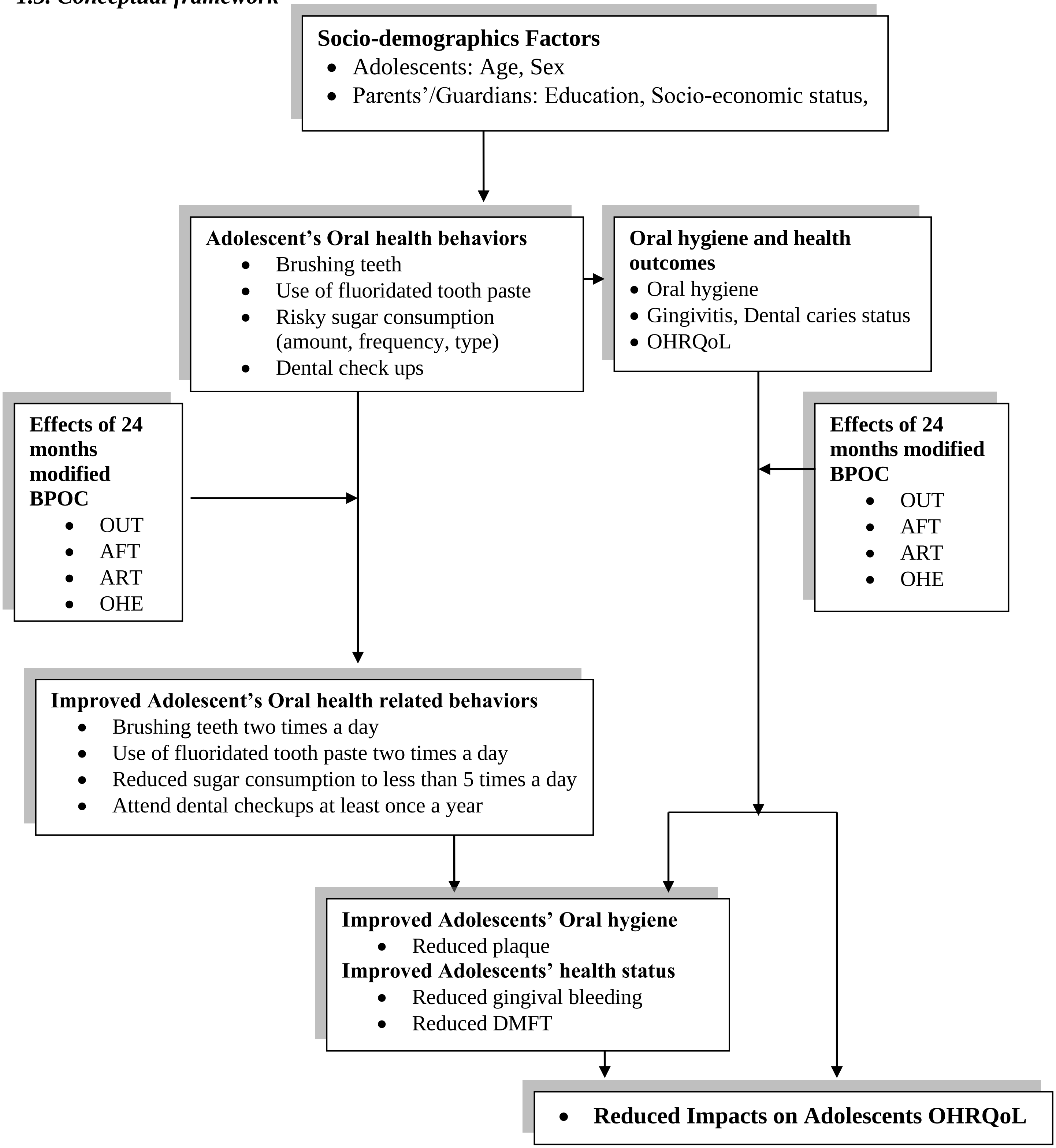

Figure 1 Conceptual framework Modified from 2002 WHO Risk factor model for promotion of oral health (Petersen, P.E., 2005)

Adolescent's demographics (age and sex) and parental/guardians (education and SES) affect oral health related-behaviors and oral health. Application of a 6 months modified BPOC will lead to an improvement in oral health-related behaviors such as brushing teeth two times a day, use of fluoridated tooth paste, reduced risky sugar consumption and dental checkups at least once a year. Improved health-related behaviors will ultimately lead to improvement in adolescent's oral hygiene and health status. Improvement in adolescent's oral hygiene and health status ultimately lead to reduced impacts in oral health-related quality of life among adolescents. The intervention can also directly improve adolescent's oral hygiene and health status and reduce impacts on adolescents OHRQoL.

1.4. Rationale

Oral health services in Zambia have been inclined towards curative rather than preventive approach for a number of decades due to lack of emphasis, planning, budgeting and funding of preventive services. The curative approach relies on inadequate number and un evenly distributed dental clinics which are mainly concentrated in urban areas. Most of the clinics render limited services mainly extraction of teeth, due to lack of enough funds to procure equipment and materials for restorative services. Accessibility and affordability of available curative services is a challenge especially among rural communities which may potentially create oral health inequalities. Word Health Organization (WHO) recommended Basic Package of Oral Care (BPOC) as an easy to conduct, cheap, and effective strategy in prevention and management of oral diseases in low to middle income countries. Despite of potential benefits the strategy has not been tested and used in most countries including Zambia. The shortfall could be explained by inadequate advocacy of importance of preventive over curative oral health services during training and oral health services delivery, inadequate planning and budgeting for oral health research and preventive services, and lack of oral health data compared to other NCDs in some African countries including Zambia. One of the targets of 2017-2021 Zambia National Health strategic plan is to strengthen and orient health systems to address the prevention and control of NCDs and the underlying social determinants through people-centered primary health care and Universal Health Coverage. This goal

cannot be realized without evidence-based results of the magnitude of non-communicable disease in particular oral diseases and effectiveness of preventive strategies such as WHO-BPOC. This study will highlight the magnitude of dental caries and gingivitis among adolescents and effectiveness of WHO-BPOC strategy in oral diseases prevention. The data will help oral health planners to convince policy makers and planners at ministry of health to plan for preventive oral health services in the country. The study will also improve training of the fifty-year bachelor of dental surgery students who will be involved during execution of BPOC components (OUT, AFT and ART) under supervision. The training will not only impart knowledge on prevention to the dental students but also orient them towards diseases prevention. The study will also contribute towards realizing the 2017-2021 strategic plan goal of creating sustainable and peoples – centered primary health care by empowering students and teachers assigned to the project with knowledge to give oral health education.

1.5. Research hypothesis and Questions

1.5.1 Hypothesis

Null hypothesis

Curative and preventive measures given through a modified WHO -BPOC has no effect on oral health knowledge and related behaviors, quality of life and socio-demographic gradient in oral health of adolescents in Copperbelt province, Zambia.

Alternative hypothesis

Curative and preventive measures given through a modified WHO -BPOC has effect on oral health knowledge and related behaviors, quality of life and socio-demographic gradient in oral health of adolescents in Copperbelt province, Zambia.

1.5.2 Research questions

1. What is the extent of poor oral hygiene, gingivitis, dental caries, and OHRQL among adolescents in Copperbelt province, Zambia?
2. What are the determinants of poor oral hygiene, oral health and OHRQoL among adolescents in Copperbelt province, Zambia?
3. Does modified –WHO Basic Package of Oral Health Care (BPOC) have effect on improving oral health, related behaviors and OHRQoL among adolescents in Copperbelt province, Zambia?
4. Does modified –WHO Basic Package of Oral Health Care (BPOC) have effect on reducing socio-demographic gradient in oral health, related behaviors and OHRQoL among adolescents in Copperbelt province, Zambia?

2.0. Objectives

2.1. Broad Objective

To evaluate the effectiveness of applying the modified WHO basic package of oral care (BPOC) in improving oral health knowledge and related behaviors, oral health and OHRQoL among adolescents in Copperbelt, Zambia

2.2. Specific Objectives

1. To determine prevalence and distribution of dental caries, periodontal conditions and oral impacts on daily performance among adolescents in Copperbelt Province, Zambia
2. To determine the association between dental caries and oral health related quality of life among adolescents in Copperbelt Province
3. To assess the effects of a six months modified basic package of oral care intervention on knowledge and behaviors related to dental caries after 18 and 24 months of follow up.
4. To assess the effects of a six months modified basic package of oral care intervention on prevalence of dental caries after 18 and 24 months of follow up
5. To assess the effects of a six months modified basic package of oral care intervention on prevalence of oral impacts on daily performance after 18 and 24 months of follow up

3.0. Literature review

Occurrence of dental caries, periodontal conditions and oral impacts on daily performance among adolescents

Dental caries affect about 19-93.7% of adolescents globally and its distribution varies by demographic and social factors (Aldosari et al., 2004, Mtaya et al., 2009, Nurelhuda et al., 2009, Mashoto et al., 2010, Singh., 2011, Pakpour et al., 2011, Krisdapong et al., 2013, Al-Darwish et al., 2014, Mathur et al.,2014, Dye et al., 2015, Vernazza et al., 2016, Msyamboza et al., 2016, Lage et al., 2017, Borges et al., 2017, Giacaman et al, 2018, Simangwa et al., 2018, Simushi et al., 2018, Clark et al., 2019). The prevalence ranges from 41-72% in Europe and America (Jamelli et al., 2010, Dye et al., 2015, Lyra et al.,2015, Vernazza et al., 2016, Borges et al,. 2017, Lage et al,. 2017, Giacaman., 2018), 20.0-93.7% in Asia (Al- Sadhan, S., 2006, Pakpour et al., 2011, Krisdapong et al.,2013, Mathur et al.,2014, Al-Darwish et al,.2014,) and 8.8%- 50.98% in Africa (Nurelhuda et al., 2009, Mashoto et al,. 2010, Singh., 2011, Msyamboza et al., 2016, Simagwa et al,. 2018, Simushi et al,. 2018). While some studies in Europe and America report no significant difference in caries occurrence by age and sex (Dye et al., 2015, Borges et al., 2017, Lage et al., 2017, Clark et al., 2019), others report significantly more girls than boys (Vernazza et al., 2016) and older than younger adolescents (Dye et al., 2015, Lyra et al., 2015) are affected. The disease also significantly affects more adolescents in rural than urban areas (Lyra et al., 2015, Borges et al., 2017, Giacaman., 2018) and those from larger than smaller family sizes (Lyra et al., 2015). In Africa and Asia, the disease affects significantly more girls than boys (Al-Sadhan, S., 2006, Al-Darwish et al,.2014, Msyamboza et al., 2016,), older than younger adolescents (Al-Darwish et al,.2014, Simagwa et al,. 2018), and those from lower than higher socioeconomic status (Nurelhuda et al., 2009, Pakpour et al., 2011, Mathur et al.,2014, Krisdapong et al., 2013). The disease also affect significantly more urban than rural adolescents (Nurelhuda et al., 2009, Owino et al., 2010, Simushi et al,. 2018), however Mashoto et al., (2010) and Mapengo et al., (2010) reported the opposite and Msyamboza et al., (2016) reported no difference.

Proportion of adolescents with plaque ranges from 3.5-27.2% in Europe and America (Vadiakas et al., 2012, Botero et al.,2015, Giacaman et al., 2018) and 16.1-81.1% in Asia and Africa (Yazdani et al., 2009, Mbawala et al., 2010, Berhane et al., 2014, Sukhabogi et al., 2014, Oyedele et al.,2018, Simangwa et al., 2018). The proportion is significantly higher in older, males, and those from rural and low socioeconomic status in all regions (Mbawala et al., 2010, Vadiakas et al., 2012, Oyedele et al.,2018, Simangwa et al., 2018, Giacaman et al., 2018). Plaque is also reported in Africa and Europe to be more among adolescents from lower than higher fathers or mother's education (Mbawala et al., 2010, Vadiakas et al., 2012, Berhane et al., 2014).

Prevalence of gingivitis among adolescents range from 16.7-72.8% in Europe and America (Vadiakas et al., 2012, Botero et al., 2015, Chyrsanthakopoulo et al., 2020), 59-100% in Asia and middle east (Sharva et al., 2014, Sukhabogi et al., 2014) and 26.7-40.9% in Africa (Azodo et al., 2015, Simangwa et al., 2018). Gingivitis affects significantly more adolescents in rural than urban areas, males than females (Vadiakas et al., 2012, Nuaimi et al., 2014, Simangwa et al,. 2018), those from low socio-economic status families and parents with low education (Mbawala et al., 2010, Vadiakas et al., 2012, Sharva et al., 2014) in all regions. Information on clinically assessed gingivitis among Zambian adolescents could not be retrieved, however a self reported experience of gingivitis is 19% (Hagberg and Sjodahl 2007).

Impact of oral diseases on daily performance is reported by 38.5-88.7% of adolescents in Europe and America (Bianco et al., 2010, Cortes-Martinicorena et al., 2010, Castro et al., 2011), 44-89.8% in Asia (Usha et al., 2011, Krisdempong et al., 2013, Amilani et al., 2020), 28.6–56.5% in Africa (Mtaya et al., 2007, Nurehulda et al., 2010, Chukumwa et al., 2016). The proportion of Zambian adolescents reporting oral impacts on daily performance vary widely from 11.7- 61.5% (Andersson et al,. 2017, Anthony et al,. 2018). Oral impacts are reported more by girls than boys, those from higher than lower socio-economic status, and urban than rural adolescents (Mtaya et al., 2007, Mashoto et al., 2009, Nurehulda et al., 2010, Mbawala et al., 2011, Chukumwa et al., 2016, Anthony et al,. 2018). Difficulties on eating and cleaning mouth are the commonly reported impacts (Mtaya et al., 2007, Mashoto et al., 2009., Nurehulda et al., 2010, Bianco et al., 2010, Cortes-Martinicorena et al., 2010, Castro et al., 2011, Mbawala et al., 2011, Usha

et al., 2011, Chukumwa et al., 2016). Additionally, difficult in smiling and emotion disturbances are mostly reported impacts among European and American adolescents (Bianco et al., 2010, Cortes-Martinicorena et al., 2010, Castro et al., 2011) while speaking difficulties is reported in Africa and Asia (Mtaya et al., 2007, Usha et al., 2011).

Available information on oral disease occurrence in terms of magnitude and distribution among African adolescents was published about a decade ago. The prevailing changes in lifestyles make these disease states not suitable for extrapolation in Zambian adolescents to sufficiently inform the government in making appropriate oral health plans for this group.

Adolescent's oral health knowledge and related behaviours

The proportion of adolescents with knowledge on sugar containing food as a cause of tooth decay is reported to range from 50-86.5% in Europe and America (Smyth et al., 2007, Graça et al., 2019), 27.5-69.4% in Asia (Lian et al., 2010, Dixit et al., 2013, Hague et al., 2016) and 48.2%-94.6% in Africa (Oredugba et al., 2004, Mashoto et al., 2009, Okemwa et al., 2010, Mbawala et al., 2010, Nyamuryekung'e, 2012). The proportion of those with knowledge on the importance of regular tooth brushing ranges from 19-98.1% in all regions (Oredugba et al., 2004, Shenoy et al., 2010, Dixit et al., 2013, Hague et al., 2016, Graça et al., 2019). It is 33-71% in Europe and America (Smyth et al., 2007, Graça et al., 2019), 19.0-57.4% in Asia (Lian et al., 2010, Shenoy et al., 2010, Dixit et al., 2013) and 65-98.1% in Africa (Darout et al., 2005, Carneiro et al., 2011, Varenne et al., 2006). Percentage of adolescents with knowledge on use of fluoridated toothpaste as a means of preventing dental caries ranged from 40.5-75.3% in Europe and America (Smyth et al., 2007, Graça et al., 2019), 18-83.7% in Asia (Lian et al., 2010, Dixit et al., 2013, Sanadhya et al., 2014, Hague et al., 2016) and 6-87.7% in Africa (Varenne et al., 2006, Carneiro et al., 2011, Elzahaf et al., 2019). Percentage of adolescents with knowledge on importance of dental check up at least once a year range as follows 35.4-50% in Europe and America (Smyth et al., 2007, Graça et al., 2019) and 42-98.1% in Asia and Africa (Blaggana et al.,2016. Al-Hussaini et al.,2003, Carneiro et al., 2011, Varenne et al., 2006)

The proportion of adolescents practicing good oral health related behaviours in Europe and America are; 62-100% for regular tooth brushing (Ericsson et al., 2012, Smyth et al., 2007, Veiga et al., 2014, Bombert et al., 2018, Graça et al., 2019), 4.-14.4 % for flossing (Ericsson et al., 2012, Veiga et al., 2014, Bombert et al., 2018, Graça et al., 2019) 26-41% for sugar consumption of less than five times a day (Smyth et al., 2007,Graça et al., 2019), 39.2.-75% for dental check up at least once a year (Vadiakas et al., 2012, Veiga et al., 2014, Bombert et al., 2018, Graça et al., 2019) and more than 90% report to use fluoridated toothpaste (Bombert et al., 2018, Graça et al., 2019). In Asia 37.5-95.7% of adolescents brush their teeth regularly (Zhu et al., 2003, Lian et al., 2010, Hague et al., 2016, Asubaie et al., 2019), 0-6.6% floss (Pakpour 2011, Subaie et al., 2019), 9.3%-24.9% consume sugar containing foods less than five times a day (Zhu et al., 2003, Lian et al., 2010), 36.6-43.9% attend dental check up at least once a year (Zhu et al., 2003, Pakpour 2011, Asubaie et al., 2019) and 4.5-89.8%. to use fluoridated toothpaste (David et al., 2005, Bhardwaj et al 2013., Hague et al 2016) and only 12.7% change tooth brushes after every three months or when bristles flair (Hague et al 2016). In Africa, 62.2-92% of adolescents are reported to brush their teeth regularly (Oredugba et al., 2004, Mbawala et al., 2010, Okemwa et al., 2010, Kahabuka et al., 2018), 0.3 -11.3% to floss (Carneiro et al., 2011, Azodo and Agbor 2015, Elzahaf et al.,2019), 38.8-91.3% to consume sugar containing foods less than five times a day (David et al 2005, Carneiro et al., 2011, Kahabuka et al., 2018), 12.6-.53% attend for dental check up at least once a year (Oredugba et al., 2004, Mbawala et al., 2010, Kahabuka et al., 2018) and 38.9-81.3% use fluoridated toothpaste (Okemwa et al., 2010, Elzahaf et al.,2019). Good adolescents' oral health related behaviours are reported significantly more in females than males (Okemwa et al., 2010, Carneiro et al., 2011, Bombert et al., 2018) older than younger (Kahabuka et al., 2018), those from urban than rural, from higher than lower socio-economic status families (Mashoto et al., 2010, Bombert et al., 2018, Kahabuka et al., 2018) and whose parents have higher than lower education level (Kahabuka et al., 2018, Bombert et al., 2018). Information on oral health knowledge and behaviors among Zambian adolescents is scarce. Only McKittrick and Jacobsen, (2014) and Hamonga et al., (2015) reported 10% and 71.9% as the proportion of adolescents practicing tooth

brushing twice a day or more respectively. Other elements of knowledge and behaviors could not be retrieved.

Effects of Oral Health Education (OHE) on adolescents' oral health knowledge and related behaviours

Improvement in knowledge following OHE has been reported to be; 46-100% for tooth brushing (D'Cruz et al.,2013, Sanadhya et al., 2014, Hague et al., 2016, Vangiparum et al.,2016, Naidu et al., 2017), 40.1-98.9% for sugar containing food/drinks as cause of tooth decay (Sanadhya et al., 2014, Hague et al., 2016, Naidu et al., 2017) and 31.5-52.2% on the role of fluoridated tooth paste (Sanadhya et al., 2014, Hague et al., 2016).

Improvement in tooth brushing frequency following OHE is reported to range from 23 to 47% (Haque *et al.*, 2016; Pakpour *et al.*, 2016; Melo *et al.*, 2018, Esan et al,. 2015, Åstrøm et al 2012), reduction in risky sugar consumption 28.8-31% (Haque *et al.*, 2016 , Naidu and Nandlal, 2017 Esan et al., 2015), regular use of fluoride toothpaste 26-42% (Melo *et al.*, 2018, Wickreasinghe and Ekanayake, 2017, Hague et al., 2016) and 26% for regular preventive dental check-ups (Wickremasinghe and Ekanayake, 2017). However, other researchers found no statistical significant improvement in; regular dental visit (Dudovitz *et al.*, 2018), reduction in risky sugar consumption (Wickremasinghe and Ekanayake, 2017, Dudovitz *et al.*, 2018) and regular use of fluoride toothpaste (Yang *et al.*, 2009).The available information is inconclusive with some studies reporting improvement in adolescents' knowledge and behaviours following OHE intervention while others not.

Effects of Oral Health Education (OHE) on adolescents' plaque score

Six months to three years OHE interventions on reducing plaque have been conducted using different methods such as classroom sessions and demonstrations (Yekaninejad *et al.*, 2012, Haleem et al,.2012, Reddy *et al.*, 2016), supervised tooth brushing (Hilgert *et al.*, 2015) or combined strategies. A wide range of improvement (8 to 67%) in adolescents' plaque score following OHE has been reported (Haleem et al,.2012, Yekaninejad *et al.*, 2012, D'Cruz and Aradhya, 2013, Hilgert *et al.*, 2015, Reddy *et al.*,

2016, Naidu and Nandlal, 2017) though other authors did not find significant improvement (Frencken et., al 2001, Mbawalla et al., 2012). The effectiveness of OHE in plaque score reduction was reported by Hallem et al., (2016) to be influenced by mode of delivery, repetitive sessions and reinforcement of messages. Observed wide range of effect of OHE on plaque score may have been due to lack of consistence on study duration, mode of oral health delivery and intentional planned OHE intervention activities.

Effects of Oral Health Education (OHE) on adolescents' gingival bleeding score

Different OHE methods such as classroom sessions and demonstrations (Mbawala et al., 2012, Yekaninejad *et al.*, 2012, Gauba et al 2013) supervised tooth brushing (Hilgert *et al.*, 2015) or combined strategies have been used to deliver OHE and resulted in reduction of gingival bleeding among adolescents. The follow up duration varied from 3 weeks to 2 years. The interventions achieved 10.3-50% reduction in the proportion of adolescents with gingival bleeding (Biesbrock et al., 2003, Ganesh et al.,2007, Mbawala et al., 2012, Yekaninejad *et al.*, 2012, D'Cruz and Aradhya, 2013, Gauba et al 2013) Reduction in bleeding score is reported significantly more in females than males and urban than rural adolescents (Mbawala et al., 2012). Most OHE messages used in the cited literature did not specifically target prevention of gingivitis. Additionally, socio-demographic differences in gingival bleeding reduction was not assessed by most authors.

Effects of BPOC on adolescents' dental caries status

Oral Health Education interventions intending to reduce dental caries with duration of 6 months to 3.5 years have been conducted (Frencken et al., 2001, Mbawala et al., 2012, Haque *et al.*, 2016, Stein *et al.*, 2018). Haque *et al.*, (2016) and Qudri et al., (2018) report 25% and 35% respectively, reduction in dental caries following OHE while others did not find conclusive evidence to support effectiveness of OHE in reducing dental caries.

Tooth brushing interventions with a follow up time ranging from 6 months to 3 years have been conducted through supervised (Hilgert *et al.*, 2015, Petersen *et al.*, 2015, Clark *et al.*, 2019) and unsupervised methods (Marinho et al., 2003, Kumar et al., 2016, van der

Walt et al., 2018). The inteventions reduced the proportion of adolescents with dental caries in a range of 24-50%. Frequency of toothpaste use is reported to influence the effectiveness of fluoride toothpaste in caries prevention. A systematic review reports an average of 24% reduction in decayed, missing and filled tooth surface as a result of use of fluoridated tooth paste (Marinho et al., 2003). No age and sex differences in caries reduction are reported (Clark *et al.*, 2019). No information was accessed where both OHE and fluoride toothpaste were used as a package in caries prevention interventions.

Effects of BPOC on adolescents' OHRQoL

No information reporting effect of BPOC as a package on adolescents' OHRQoL could be accessed. Available studies reporting effects on adolescents' OHRQoL used OHE, ART and/or OUT. Applying ART and OUT to school adolescents, Mashoto et al,. (2010) reported 13.7% improvements in overall adolescents' oral impacts with minimal effect size in eating speaking, cleaning, smiling, emotion disturbance, school work and social role. Whereas Chukwumah et al., (2016) in a dental facility-based study reported an overall marked improvement in oral impacts; the effect size was moderate to large for all domains ranging from 0.2 for smiling and participating in social role to 0.9 for eating. No significant differences in oral impacts improvement by age, sex or socio-economic status was reported (Chukwumah et al., 2016).

Effects of modified BPOC on adolescents' socio-demographic differences in oral health status and OHRQoL

Pattussi et al. (2006) and Mbawalla et al,. (2012) reported that provision of oral health education achieved reduction of inequalities in dental caries and gingival health among adolescents of Brazil and Tanzania, respectively. However, Qadri *et al.*, 2018 reported widening of socio-economic disparity in caries; caries reduction being more among adolescents from high SES families while other investigators state that the effect of OHE in reducing oral health inequalities is inconclusive (Polk et al., 2010, Roncalli *et al.*, 2015). No findings could be accessed reporting changes of socio-demographic inequalities in oral health status and related quality of life following ART and OUT.

4.0. Materials and Methods

4.1. Study design

This will be a cluster randomized controlled field trial to assess the effectiveness of applying the WHO basic package of oral care (BPOC) and oral health education in improving oral health knowledge and related behaviors, oral health and OHRQoL among adolescents in Copperbelt, Zambia**.**

4.2. Study area

The study will be conducted in Copperbelt province, the second highly populated province in Zambia after Lusaka with a total population of 2,542,132 out of which 24% are adolescents. Administratively the province is divided into 10 districts with a total number of 102 government and private secondary schools located in both rural and urban set up. Copperbelt residents engage in different economic activities such as mining and quarrying, light industry manufacturing, animal keeping and small- and large-scale farming which can account for differences in socio-economic status. Socio-demographic mix of adolescents in the Copperbelt province provides a typical representation of Zambian adolescents and therefore enables inference of the results to the whole country at large.

4.3. Study population

The study will involve adolescents aged 10-14 years in their first year of secondary school in Copperbelt Province, Zambia. Adolescence is an important phase of life which marks transition from childhood to adulthood and oral health related behaviors acquired during this phase affect their current and future oral health. Adolescents' oral health status and related behaviors are directly dependent on their family's socio-economic status and therefore the effect of any socioeconomic gradient in health is likely to be reflected in this group.

4.4. Sample size

A sample size of 1760 adolescents calculated using a Cluster Randomized Controlled Trial formula (RCT) and adjustment to account for design effect and 10% loss to follow up as shown below is expected to be sufficient for the study.

(i) Sample size formula for RCT considering simple random sampling:

$$2N = \frac{4(Z_{\alpha} + Z_{\beta})^{2}\, \sigma^{2})}{\delta^{2}}$$

Where

2N is number of participants in two arms

Z_{α} is point on standard normal distribution corresponding to level of confidence of the study estimated to be 1.96 for 95% Confidence Interval

Z_{β} is value corresponding to 85% power of the study =1.03

σ is known mean DMFT from a previous study in Ndola, Zambia by Nasilele et al (2018)=1.34

δ is the expected clinically significant change in mean DMFT set at 20% =0.268

Based on assumptions and known values above the sample size for 2 arms is calculated as follow:

$$2N = \frac{4(1.96+1.03)^{2}\,(1.34)^{2}}{(0.268)^{2}}$$

2N= 894.01=894

(ii) Sample size considering cluster design, hence adjusting for design effect

$nc = n_1[1 + (m - 1)\,\rho\,]$

nc $=894[1 + (80 - 1)\ 0.01]$

nc =estimated to be 1600.26=1600

Where

n_c = Number of pupils/participants in cluster per arm

n_1 = Individual number of participants (pupils) in an arm when considering random sampling

ρ = Estimated intra-cluster correlation coefficient (ICC) to be 0.01

m = Estimated cluster size in this study is 80 students in a class

(iii) Sample size adjustment to consider loss to follow up assumed at 10%

nc = 1600 +{1600 x(10/100)} =1760

Final minimum sample size considered = 1760

4.5. Sampling

A two-stage cluster sampling technique will be used; district being a sampling unit at stage one and secondary schools at stage two. Through simple random sampling, three out of ten districts of Copperbelt province will be selected and from each district a total of six clusters (secondary schools) will thereafter be selected as part of the study. To attain a sample size of 1,760 participants, 18 clusters with cluster size of 90-100 will be involved. All adolescents aged 10-14 in grade 8 at selected clusters will be eligible to participate in this study.

4.6. The trial intervention

The trial intervention is a modified WHO –BPOC which include all the 3 packages of BPOC plus oral health education. The first package, Oral Urgent Treatment (OUT) will include simple extraction of teeth beyond restorable condition and referral for teeth requiring clinic set up for removal. The second package which is Atraumatic Restorative Treatment (ART) will be executed using hand instruments only by removing decayed part of teeth with cavities not reaching the pulp or not diagnosed as irreversible pulpitis then filling with Fuji IX Glass- ionomer. The third component (Affordable Fluoridated Tooth pastes) will be done by providing 250g pack of fluoride toothpaste every two months and toothbrushes every three months for 18 months. In addition to WHO-BPOC the study will also give peer provided oral health education. The peer educators will be furnished with the following printed messages to deliver to their classmates in the intervention groups once after every two weeks for a period of 18 months;

1. Restrict the frequency of taking sugary food and drinks in the diet to less than five times per day.
2. Brush your teeth for 2 minutes ensuring all the surfaces are cleaned; twice per day in the morning and evening before retiring to bed.
3. Use fluoride toothpaste to brush, spit the foam but do not rinse it out.
4. Change your toothbrush every 3 months or when bristles flare out.
5. Advise your parent or guardian to buy a tooth paste containing at least 1450 ppm fluoride and to take you for dental check up at least once a year.

Table 1 Summary of intervention set up

Baseline data collection	Randomization	Activities in each group	Activity Schedule Months									
			1-2	3-4	5-6	7-8	9-10	11-12	13-14	15-16	17-18	19-24
• OHK<br>• OHRB<br>• Plaque<br>• Gingival bleeding<br>• Dental caries	Intervention	1. Peer led OHE	OHE once after every 2 weeks (total 36 sessions)									-No OHE
		2. OUT sessions 1-3	1st					2nd			3rd	No OUT
		3. ART sessions 1-3	1st					2nd			3rd	NO ART
		4. AFT sessions 1-9	1st	2nd	3rd	4th	5th	6th	7th	8th	9th	-No AFT
		5. Follow ups (at 3, 6, 12, 18, and 24 months)			1st			2nd			3rd	4th
	Control	1. Modified BPOC (OHE/OUT/ART/AFT) at the end										B<br>P<br>O<br>C
		2. Routine oral care at home	Continue with standard oral care at home									
		3. Follow ups			1st			2nd			3rd	4th

4.6.1. Intervention groups

The trial will have one intervention and a control group. The intervention group will receive a modified WHO-BPOC at school and the control will continue with their daily standard of care during the study period.

4.6.2. Intervention period

The intervention will be instituted immediately after base line data collection and randomization and will be for a period of 24 months follow up.

4.7. Randomization

Allocation of clusters (secondary schools) into intervention and control groups will be done by stratified block randomization procedure whereby the district will be a stratifying unit and schools a blocking unit. Four randomly selected schools in the three districts will be listed in alphabetical order and assigned numbers 1 to 4. Computer random number generator will be used to randomly allocate 2 clusters in intervention and 2 in control groups in each district. The first two numbers generated will be allocated to intervention and the second two in control.

4.8. Inclusion and exclusion

4.8.1. Cluster level

Inclusion criteria

- Secondary within Copper belt providence with grade 8 students

Exclusion criteria

- Schools involved in any other oral health intervention programs
- Boarding schools
- Single sex schools
- Special need schools

4.8.2. Individual level

Subjects will be recruited at their respective schools during baseline data collection.

Inclusion criteria

Adolescents studying in grade 8 at schools

Exclusion criteria

Adolescents with the following conditions will be excluded

- anodontia,
- severe dental fluorosis,
- Those who have received orthodontic treatment
- With special needs.

4.9. Data collection procedures

4.9.1. Data collection tools and indices

Data will be collected using self-administered structured questionnaire and oral clinical examination. The questionnaire (Appendix 1) will be in English and will inquire on; demographic information of the participant's oral health knowledge and related behaviors and impact of oral diseases on daily performance of the adolescent. It will further inquire on parental/guardian socio-economic status using international wealth index (Smits and Steendijk, 2015). The students will understand the questions for the reason that they are fluent in English language.

Oral clinical examination will be done in a well illuminated classroom by two trained and calibrated dentists. Examination will be done using dental explorer aided by a mouth mirror and artificial light. The participants' will be lying on a desk and the examiner seated behind the participant's head. Every examiner will have one recorder who will be seated close to the examiner to ensure that the findings are recorded correctly.

Data collection will be done in four phases; baseline, every six months after institution of intervention that is; first follow up (6 months), second follow up (12 months), third follow up (18 months) and last follow up (24 months). The data collected for every adolescent will be paired and analyzed in pair. The principal investigator will assign a range of numbers for each school to be used as adolescent's identity number for baseline and all follow ups. School teachers will then be requested to create a list of names according to attendance register of that school and assign numbers to the individual participants. A copy of list of names and ID numbers will be kept by head teacher in case the teacher responsible for health is absent. Data for each variable will be entered and analyzed as set (baseline, 1st , 2nd , 3rd and 4th follow up).

4.9.2. Blinding

Single blinding will be done whereby the examiners (2 dental surgeons) will not be aware of group allocation. Blinding of the participants and investigator will not be feasible as the principal investigator will train the peers to give oral health education and be engaged in distribution of oral health materials and treatment. The participants in intervention group will also receive peer delivered oral health education and treatments therefore they will know their group.

4.10. Study outcomes

The expected primary outcomes are improved oral health (dental caries status and gingivitis) and OHRQoL. The secondary outcomes include improved oral hygiene (plaque score) and oral health knowledge and related behaviors. The effect sizes for improvement in oral health and oral health related quality of life are based on average improvement achieved in similar studies reviewed.

4.11. Study Variables

4.11.1. Independent variables

Independent variables will include demographic variables of the adolescents (age and sex) and socio-demographics of the parent/guardian (father's education, mother's education, family size, number of children, and family material possession).

Socio-demographics of the parents or guardians will be assessed by four items;

1. Education of adolescent's father and mother will be assessed as the highest level of education attained and coded as {1=No formal education, 2=Primary education, 3= Secondary education, 4= College/University, 5=I don't know, 6= Not applicable}
2. Family size will be recorded as absolute total number of people in the participant's house including parents, children and other dependants constantly living with them (not including visitors) {1 up to the highest}
3. Number of children in the family will be recorded as absolute total number of children respectively in the participant's family {1 up to the highest}
4. Material possession will be assessed using international wealth index (Smits et al., 2015).

4.11.2. Dependent variables

The dependent variables to be evaluated will include oral health related behaviors (frequency of tooth brushing, use of fluoride tooth paste, frequency of sugar consumption, dental visits), oral health outcomes (plaque, gingival bleeding, dental caries status) and OHRQoL.

4.9.2.1. Oral health related behaviors

Adolescents will be requested to report their oral health related behaviors. In respect to tooth brushing, use of fluoridated tooth paste and sugar consumption they will be required to reflect their conduct in the past 30 days. Frequency of tooth brushing per day and use of fluoridated toothpaste will be assessed and recoded using a 4-point scale follows {1= I didn't, 2= I did but not every day, 3=I did once a day, 4 = I did twice a day or more}. Intake of sugar containing drinks or foods will be assessed and recorded using a 5-point scale as follows {1= I didn't take, 2= occasionally per week, 3= Once per day, 4= Twice to four times per day, 5 = Five times or more per day}. Visiting a dentist in past one year for checkup will be assessed and recorded based on a 3-point scale {1= I didn't attend, 2= I attended once, 3=I attended twice or more}

4.9.2.2. Oral health related Quality of Life

Oral Health Related Quality of Life (OHRQoL) will be assessed using Child –OIDP tool where the participants will be asked to rate how often in the past 30 days they had impact on their daily performance (eating or enjoying food, speaking or pronouncing words, cleaning teeth, sleeping and relaxing, smiling, laughing, showing teeth without embarrassment, enjoying contact with people, maintaining usual emotional state without being irritable, carrying out school activities such as attending class or social role) due to oral diseases and scored as follows: {1=Never, 2= Once or twice a month, 3= Once or twice a week, 4= Every or nearly every day}.

4.9.2.3. Dental plaque

Will be evaluated as a sticky colorless or pale yellow to brown biofilm on tooth surface (buccal and lingual) of the index teeth (16, 11, 26, 36, 41, 46) and scored based on to Silness-Löe plaque index as follows:

0 = Plaque not visible nor cannot be wiped off with the explorer
1 = Plaque not visible but can be wiped off with the explorer
2 = Plaque is visible along the gingival no need to probe
3 = Thick plaque is visible along gingival margin

4.9.2.4. Gingival bleeding

Will be evaluated as bleeding on gingival on the index teeth (16, 11, 26, 36, 41, 46) and scored according to Silness-Löe index as follows:

0 = No inflammation (Normal gingiva, no discoloration, no bleeding)

1 = Mild inflammation (slight color change, mild alteration of gingival surface, no bleeding)

2 = Moderate inflammation (erythema, swelling, bleeding when pressure is applied)

3 = Severe inflammation (severe erythema and swelling and tendency towards spontaneous bleeding

4.9.2.5. Dental caries

Will be evaluated as any visible change of enamel color or translucent to extensive distinct cavity with visible dentin or pulp cavity and coded according to Caries Assessment Spectrum and Treatment (CAST) as follows:

Characteristic	Code	Description
Sound	0	No visible evidence of a distinct carious lesion is present
Sealant	1	Pits and/ or fissure are at least partially covered with a sealant material
Restoration	2	A cavity is restored with an (in)direct restorative material
Enamel	3	Distinct visual change in enamel only; a clear caries-related discoloration is visible, with or without localized enamel breakdown
Dentine	4	Internal caries-related discoloration in dentine; the discolored dentine is visible through the enamel, which may or may not exhibit a visible localized breakdown
	5	Distinct cavitation into dentine; the pulp chamber is intact
Pulp	6	Involvement of the pulp chamber; distinct cavitation reaching the pulp chamber, or only root fragments are present
Abscess/ fistula	7	A pus-containing swelling or a pus-releasing sinus tract related to a tooth with pulpal involvement
Lost	8	The tooth has been removed because of dental caries
Other	9	Does not match with any of the other descriptions

Investigation tools validity and reliability

4.11.3. Validity of data

Questionnaire -Questions have been adopted from standardized instruments which have been tested within the Sub-Saharan region

WHO Global school-based student health survey (WHO, 2013)

i. C-OIDP-measures of oral health quality of life (Slade, G.D., 1997),
ii. Socio-economic status - international wealth index (Smits and Steendijk, 2015)

Oral clinical examination will be done using standardized measures

i. Dental plaque Index - Sillness and Loe index (Löe, 1967)
ii. Gingival bleeding - Sillness and Loe index (Löe, 1967)
iii. Dental caries status - Caries Assessment Spectrum and Treatment (CAST) (Frencken *et al.*, 2011)

4.11.4. Reliability of data

Training and Calibration of examiners

The principal investigator (SA) is a specialist in restorative dentistry with adequate skills in dental caries diagnosis. In respect to use of Caries Assessment Spectrum and Treatment (CAST) in caries assessment the one of the inventers of the tool has provided the CAST manual and provide blended training followed by calibration. The PI will later train the two examiners (dental surgeons) on dental plaque index, gingival bleeding index and CAST for a total duration of 2 days. Training will include the rationale of using the indices their codes and description, ergonomic guidelines and instructions on how to perform examination. Pictures of clinical situations related to each code of each index will be shown. For CAST index examples of each code will be shown on extracted teeth first. The trainees will be given a number of examination exercise and their results compared to expert. Any differences will be discussed and examination repeated and in so doing the trainee will understand the tool. Training on plaque index and bleeding index will be done on adolescents with similar age group 10-14 years

Examination of 5-10 subjects aged 10-14 pre selected adolescents with full range of CAST codes will be done by each examiner and the PI then results compared. The differences will be discussed and then examination repeated. When agreement is obtained

a new set of 5-10 adolescents will be examined by trainee and results compared. Calibration will be considered sufficient when the examiners and the PI's agreement are at least 85%.

Intra-examiner consistency

The examiners will be subjected to periodic evaluation for their consistency in the clinical assessment of the indices. Every 10th participant will be subjected to a clinical examination after two weeks to test for reliability of the examiners. The coefficient of reliability will be reported as Cronbanch alpha.

4.12. Data entry cleaning and analysis

Data entry, coding and processing will be done using a computer software program IBM SPSS for Windows (Version 23). Frequency distribution for proportions, means (SD), cross tabulation with chi –square and binary logistic regression reported in odds ratio and 95% (CI) will be performed per respective objectives.

Frequency distribution for proportions of adolescents with plaque, gingival bleeding, poor oral health behaviors and oral health related quality of life. Dental caries experience will be summarized as number and percentage. Furthermore, cross tabulation for bivariate comparison of differences in intervention outcome variables by socio-demographics, study arms and oral health behaviors will be conducted. To examine for the differences between the groups (effectiveness of trial intervention) generalized estimating equations will be performed and reported in odds ratio and 95% confidence interval. A statistical significance difference shall be assumed when $p \leq 0.05$. Analysis plan per objective is summarized in appendix 6.

4.13. Ethical considerations

The ethical approval for the study will be sought from MUHAS Institutional Review Board, Tanzania and Tropical Diseases Research Control Institute (TDRC), Zambia. Permission to conduct the study shall be sought from District Education Board Secretaries (DEBS). Participation will be on voluntary bases and only adolescents whose parents will agree and sign informed written consent (Appendix 3 and 4). All treatment will be done according to Zambia dental standard treatment guidelines by qualified

registered dental personnel. Children in the control group who will be found with oral health problems will be attended and will receive the whole package of intervention at the end of the follow visit. Confidentiality of the information shall be maintained and freedom to withdraw and join the study at any time will be respected.

4.14. Study limitation and mitigation

Study limitations

1. The follow up duration will be limited to 24 months which is enough to observe change in oral health related behaviors, plaque and bleeding but not reasonably enough for observing dental caries increment.
2. The study will follow single blind design where only the examiners will be blinded, the principal investigator and the participants will not be blinded due to the nature of the intervention which requires administration of educational technical materials and provision of treatment which cannot be easily concealed.
3. Adolescents may modify their oral health related behavior in response to awareness that there are being observed (Hawthorne effects).

Mitigation

1 Dental caries assessment using CAST (Frencken *et al.*, 2011) will be used to capture incremental stages of dental caries development and dental caries variable will continue being followed up after 24 months.

2 The data analyst will be blinded during analysis stage to avoid subjective decisions on handling missing data, transforming variables, undertaking subgroup analysis, and selecting covariates due to prior knowledge of treatment allocation.

3 The interventions will end six months before the last follow up and therefore the effect of adolescents acting due to the reason that their being followed-up will be controlled in the data collected at the last follow up visit.

4.15. Dissemination of findings

Dissemination of findings will be done by presentations at regional and international conferences. The study is also expected to produce the following manuscripts for publication in local and international journals;

1. Socio-demographic and behavioral correlates of oral health among early adolescents in Copperbelt province, Zambia.
2. Impact of oral diseases on daily performance of adolescents in Copperbelt province, Zambia.
3. Impact of modified WHO –BPOC intervention on oral health and OHRQL among adolescents in Copperbelt province, Zambia.
4. Impact of modified WHO – BPOC intervention on reducing oral health disparities among adolescents in Copperbelt province, Zambia.

5.0. Budget

ITEM DESCRIPTION AND REQUIREMENTS	Unit	Quantity	Unit cost (TSh)	Total cost (TSh)
**A. Stationery and secretarial services**				
A4 sheets of papers	rim	5	10,600.00	53,000.00
Ball pens	dozen	2	5,000.00	10,000.00
Pencils	dozen	1	900.00	900.00
Note books	each	10	6,000.00	60,000.00
Printing of proposal	Copies	1	9,000.00	9,000.00
Photocopying of proposal	Copies	6	3,000.00	18,000.00
Binding of proposal	Copies	6	2,000.00	12,000.00
Consent form photocopying	Per page	1000	100.00	100,000.00
Questionnaire photocopying	Per page	5000	100.00	500,000.00
Photocopying of OHE materials	Each	120	100.00	12,000.00
Printing of thesis	Copies	1	12,000.00	12,000.00
Photocopying of thesis	Copies	4	3,000.00	12,000.00
Loose binding of thesis	Copies	4	2,000.00	8,000.00
Final binding of thesis	Copies	8	12,000.00	96,000.00
**Subtotal**				**902,900.00**
**B. Personnel costs**				
Allowance to 3 researchers	Days	90	60,000.00	5,400,000.00
Allowance to 2 assistants	Days	90	20,000.00	1,800,000.00
Incentives to teachers	Each	12	10,000.00	120,000.00
**Sub total**				**7,320,000.00**
**C. Transport and communication**				
Fuel costs (baseline data)	Liters	360	2,660.00	957,600.00
Transport Dar es Salaam to Ndola	Return air ticket	2	920,000.00	1,840,000.00
Internet and telephone	Voucher	50	5,000.00	250,000.00
**Sub total**				**3,047,600.00**

ITEM DESCRIPTION AND REQUIREMENTS	Unit	Quantity	Unit cost (TSh)	Total cost (TSh)
**D. Dental instruments, materials and supplies**				
Fuji IX	Each	12	150,000.00	1,800,000.00
Zinc oxide eugenol	Each	9	80,000.00	720,000.00
Lignocaine 2%	P/50	8	110,000.00	880,000.00
Cotton wool	500g	4	15,000.00	60,000.00
Methylated spirit	5litres	3	18,000.00	54,000.00
Sodium hypochlorite	5litres	12	8,000.00	96,000.00
Disposable cups	P/100	12	22,600.00	271,200.00
Paper towels	P/100	12	15,000.00	180,000.00
Calcium hydroxide	set	6	90,000.00	540,000.00
Tooth pastes 250mg	dozen	570	11,500.00	6,555,000.00
Tooth brushes	dozen	380	3,560.00	1,352,800.00
Examination set	set	50	60,000.00	3,000,000.00
**Sub total**				**15,509,000.00**
**E. Data Processing**				
Data entry	days	20	20,000.00	200,000.00
Data analysis	days	5	100,000.00	500,000.00
**Sub total**				**700,000.00**
**F. Dissemination**				
Attendance to local conferences	each	2	300,000.00	600,000.00
Attendance to international conferences	each	1	1,500,000.00	1,500,000.00
Publication costs	each	5	200,000.00	1,000,000.00
**Sub total**				**3,100,000.00**
**G. Other costs**				
Ethical clearance MUHAS - Tanzania	each	1	300,000.00	300,000.00
Ethical clearance TDRC - Zambia	each	1	460,000.00	460,000.00
**Subtotal**				**760,000.00**
**H. Incidental**	10%			**3,121,950.00**
**GRAND TOTAL**				**34,341,450.00**

Budget justification

A total of TSh 902,900.00 is required to facilitate purchase of stationery and payment for secretarial services. The amount will be used to print, photocopy and bind six copies research proposal. It will also facilitate printing and photocopying of consent forms, questionnaires and clinical data collection forms for 1155 participants. Furthermore, the amount will be used to print and bind 8 thesis reports. A total of TSh 7,320,000.00 is budgeted for payment of allowance for dentists and assistants who will work at the field for a total of 90 days each.

Communication and transport of researchers and assistants to and from field sites during baseline data collection, institution of the intervention and follow ups in 4 districts will require a total of TSh 3,047,600.00. Modified basic package of oral health care will involve treatment and therefore TSh 15,509,000.00 is allocated to facilitate purchase of toothpastes and brushes, dental instruments, dental materials and medical supplies. Data entry and analysis will require a total of TSh 700,000 as payment of data entry clerks and statistician. Local and international conference presentations are required as part of dissemination of findings and fulfilment of PhD and therefore TSh 3,100,000.00 is allocated as travel, accommodation and conference attendance fees.

The proposal will require to be ethically cleared by MUHAS research committee and TDRC in Zambia and for that reason TSh 760,000.00 is allocated for ethical clearance. The study will be done in span of 2 years hence 10% of the budget (TSh 3,121,950.00) is set aside to compensate for inflation and other unforeseen costs. In conclusion a total budget of **TSh 34,341,450.00** is required to facilitate completion of the research project.

.

6.0 Activity Plan

Activity		Implementation plan																								
		2020			2021									2022							2023				2024	
	Feb Jun	Jun sep	Nov	Dec	Jan-Mar	Apr	May	Jun	Jul	Aug	Sep	Oct	Nov-Dec	Jan	Feb	Mar	Apr Jun	Jul	Aug	Sep Dec	Jan-Mar	Apr-Jun	July Sept	Oct-Dec	Jan-Mar	Apr-Jun
Preparation of Research Proposal																										
Ethical approval Tanzania & Zambia																										
Training, calibration & questionnaire pretesting																										
Base line data collection & intervention																										
Attend courses MUHAS																										
Data entry, cleaning & analysis																										
Preparation of 1st manuscript																										
Submission of 1st manuscript to journal																										
1st follow up data collection (6month)																										
Data entry, cleaning & analysis																										
2nd manuscript preparation																										
Attending courses at University of Bergen																										
Submission of 2nd manuscript to journal																										

Activity	Implementation plan																								
	2020			2021									2022							2023				2024	
	Oct	Nov	Dec	Jan-Mar	Apr	May	Jun	Jul	Aug	Sep	Oct	Nov-Dec	Jan	Feb	Mar	Apr	Jun-Jul	Aug	Sep-Dec	Jan-Mar	Apr-Jun	July-Sept	Oct-Dec	Jan-Mar	Apr-Jun
Data entry, cleaning & analysis													■												
3rd manuscript preparation														■	■	■									
Submission of 3rd manuscript to journal																	■								
3rd follow up data collection (18month)																	■								
Data entry, cleaning & analysis																		■							
3rd Presentation at conferences																			■						
Writing Thesis																	■	■	■	■	■	■	■		
4th follow up data collection (24month)																					■				
Data entry, cleaning & analysis																					■				
4th manuscript preparation																						■			
4th Presentation at conferences																							■		
Submission of 4th manuscript to journal																							■		
Submission of thesis																								■	

7.0 References

Abid, A., Maatouk, F., Berrezouga, L., Azodo, C., Uti, O., El-Shamy, H. and Oginni, A., 2015. Prevalence and Severity of Oral Diseases in the Africa and Middle East Region. *Advances in Dental Research*, 27(1), pp.10-17.

Al Nuaimi, M., Ferguson, D.J. and Al-Mulla, A., 2014. Oral hygiene status in school adolescents: a study of 20,000 school-age adolescents in 66 public and private schools comparing oral hygiene status by gender and ethnicity. *South Asia*, *7781*(37.3), pp.14-06.

Al-Darwish, M., El Ansari, W. and Bener, A., 2014. Prevalence of dental caries among 12–14 year old children in Qatar.*The Saudi dental journal*,*26*(3), pp.115-125.

Aldosari, A.M., Wyne, A.H., Akpata, E.S. and Khan, N.B., 2004. Caries prevalence and its relation to water fluoride levels among schoolchildren in Central Province of Saudi Arabia.*International Dental Journal*,*54*(6), pp.424-428.

Al-Sadhan, S., 2006. Dental caries prevalence among 12–14 year-old schoolchildren in Riyadh: a 14 year follow-up study of the oral health survey of Saudi Arabia phase I. Saudi Dent. J. 18, pp2–7.

Alsubaie, A.S.R., 2019. Oral health-related behaviors and dental pain among children in Saudi Arabia. *Journal of International Oral Health*, *11*(1), pp.1.

Amilani, U., Jayasekara, P., Perera, I.R., Carter, H.E., Senanayake, S. and Kularatna, S., 2020. Oral impact on daily performance (OIDP) scale for use in Sri Lankan adolescents: a cross sectional modification and validation study.*BMC oral health*,*20*(1), pp.16.

Andersson P, J Kavakure , P Lingström The impact of oral health on daily performances and its association with clinical variables in a population in Zambia. International Jounal of Dental hygiene 2015; 15(2) pp128-134.

Anthony, S.N., Zimba, K. and Subramanian, B., 2018. Impact of malocclusions on the oral health-related quality of life of early adolescents in Ndola, Zambia. *International journal of dentistry*, *2018*.

Arcaya, M.C., Arcaya, A.L. and Subramanian, S.V., 2015. Inequalities in health: definitions, concepts, and theories. *Global health action*, *8* (1), pp. 27106.

Artemisa, M., Mapengo, A., Marsicano, J,. Avansine Moura, Patrícia G., Arsenio H., 2010. Dental caries in adolescents from public schools in Maputo, *International Dental Journal*, 60 pp. 273–281.

Åstrøm, A. N. and Mashoto, K. O. 2012 Changes in oral health related knowledge, attitudes and behaviours following school based oral health education and atraumatic restorative treatment in rural Tanzania', *Norsk Epidemiologi*, 22(1), pp. 21–30.

Azodo, C.C. and Agbor, A.M., 2015. Gingival health and oral hygiene practices of schoolchildren in the North West Region of Cameroon.*BMC research notes*,*8*(1), pp.385.

Baiju, R.M., Peter, E., Varghese, N.O. and Sivaram, R., 2017. Oral health and quality of life: current concepts. *Journal of clinical and diagnostic research 11*(6), pp.ZE21.

Batra M, Agarwal N, Singh A, Dutt M, Sinha A. 2014. Basic package for oral care: a step towards primary oral health care. *TMU Journal of Dentistry*. Apr; (1): pp 57-60.

Benzian, H., Varenne, B., Stauf, N., Garg, R., Monse, B., Bundy, D.A.P., de Silva, N., Horton, S., Jamison, D.T. and Patton, G.C., 2017. Promoting Oral Health through Programs in Middle Childhood and Adolescence. *Disease Control Priorities*, pp*8*.

Bergqvist, K., M.Å. Yngwe, and O. Lundberg, Understanding the role of welfare state characteristics for health and inequalities–an analytical review. BMC public health, 2013. 13(1): p. 1234.

Berhane, H.Y. and Worku, A., 2014. Oral health of young adolescents in Addis Ababa—a community-based study. *Open Journal of Preventive Medicine*, *2014*.pp 640-648

Bhardwaj, V.K., Sharma, K.R., Luthra, R.P., Jhingta, P., Sharma, D. and Justa, A., 2013. Impact of school-based oral health education program on oral health of 12 and 15 years old school children. *Journal of education and health promotion*, pp*2*.

Bianco, A., Fortunato, L., Nobile, C.G.A. and Pavia, M., 2010. Prevalence and determinants of oral impacts on daily performance: results from a survey among school children in Italy. *European Journal of Public Health*, *20*(5), pp.595-600.

Biesbrock AR, Walters PA, Bartizek RD. Initial impact of a national dental education program on the oral health and dental knowledge of children. J Contemporary Dentistry Practice 2003; 2: pp1–10.

Blaggana, A., Grover, V., Anjali, A.K., Blaggana, V., Tanwar, R., Kaur, H. and Haneet, R.K., 2016. Oral health knowledge, attitudes and practice behaviour among secondary school children in Chandigarh. *Journal of clinical and diagnostic research: JCDR*, *10*(10), p.ZC01.

Bombert, F., Manso, A.C., Sousa Ferreira, C., Nogueira, P. and Nunes, C., 2018. Sociodemographic factors associated with oral health in 12-year-old adolescents: hygiene behaviours and health appointments. A cross-sectional national study in Portugal. *International dental journal*, *68*(5), pp.327-335.

Borges T.S., Cardoso M.Z., Fortuna M.J., Reuter C.P., Imperatore S., Franke S.I.R. and Burgos M.S., 2017. Oral hygiene, dietary habits and prevalence of dental caries in adolescents from rural and urban areas in Rio Grande do Sul, Brazil.*RGO-Revista Gaúcha de Odontologia*,*65*(2), pp.139-147.

Botero JE, Rösing CK, Duque A, Jaramillo A, Contreras A. Periodontal disease in children and adolescents of Latin America. Periodontol 2000 2015; 67: 34-57.

Carneiro, L., Kabulwa, M., Makyao, M., Mrosso, G. and Choum, R., 2011. Oral health knowledge and practices of secondary school students, Tanga, Tanzania. *International Journal of Dentistry*, *2011*.

Castro, R.D.A., Portela, M.C., Leão, A.T. and de Vasconcellos, M.T., 2011. Oral health–related quality of life of 11-and 12-year-old public school children in Rio de Janeiro. *Community dentistry and oral epidemiology*, *39*(4), pp.336-344.

Chrysanthakopoulos, N.A., 2016. Prevalence of gingivitis and associated factors in 13-16-year-old adolescents in Greece. *European Journal of General Dentistry*, *5*(2), pp.58.

Chukwumah, N.M., Folayan, M.O., Oziegbe, E.O. and Umweni, A.A., 2016. Impact of dental caries and its treatment on the quality of life of 12-to 15-year-old adolescents in B enin, N igeria. *International journal of paediatric dentistry*, *26*(1), pp.66-76.

Clark, E., Foster Page, L.A., Larkins, K., Leon de la Barra, S. and Thomson, W.M., 2019. Caries-preventive efficacy of a supervised school toothbrushing programme in Northland, New Zealand. *Community Dental Health*.

Cortés-Martinicorena, F.J., Rosel-Gallardo, E., Artázcoz-Osés, J., Bravo, M. and Tsakos, G., 2010. Adaptation and validation for Spain of the Child-Oral Impact on Daily Performance (C-OIDP) for use with adolescents. *Med Oral Patol Oral Cir Bucal*, *15*(1), pp.e106-e111.

Curtis, A.C., 2015. Defining adolescence.Journal of Adolescent and Family Health, 7(2), p.2

Darout, I.A., Åstrøm, A.N. and Skaug, N., 2005. Knowledge and behaviour related to oral health among secondary school students in Khartoum Province, Sudan.*International dental journal*,*55*(4), pp.224-230.

David, J., Wang, N.J., Åstrøm, A.N. and Kuriakose, S., 2005. Dental caries and associated factors in 12-year-old schoolchildren in Thiruvananthapuram, Kerala, India. *International journal of paediatric dentistry*,*15*(6), pp.420-428.

D'Cruz, A.M. and Aradhya, S., 2013. Impact of oral health education on oral hygiene knowledge, practices, plaque control and gingival health of 13-to 15-year-old school children in Bangalore city. *International journal of dental hygiene*, *11*(2), pp.126-133.

Dixit, L.P., Shakya, A., Shrestha, M. and Shrestha, A., 2013. Dental caries prevalence, oral health knowledge and practice among indigenous Chepang school children of Nepal. *BMC oral Health*, *13*(1), pp.20.

Dudovitz, R.N., Valiente, J.E., Espinosa, G., Yepes, C., Padilla, C., Puffer, M., Slavkin, H.C. and Chung, P.J., 2018. A school-based public health model to reduce oral health disparities. *Journal of public health dentistry*, *78*(1), pp.9-16.

Dye BA, Thornton-Evans G, Li X, Iafolla TJ. Dental caries and sealant prevalence in children and adolescents in the United States, 2011-2012. NCHS Data Brief 2015;(191):pp1-8.

Elzahaf, R.A., Elzer, A.S. and Edwebi, S., 2019. Oral health practices, knowledge, and attitudes among primary schoolchildren in Derna City, Libya: A cross-sectional survey.*International Journal of Pedodontic Rehabilitation*,*4*(2), pp.41.

Ericsson, J.S., Östberg, A.L., Wennström, J.L. and Abrahamsson, K.H., 2012. Oral health-related perceptions, attitudes, and behavior in relation to oral hygiene conditions in an adolescent population. *European journal of oral sciences*, *120*(4), pp.335-341.

Esan, A., Folayan, M.O., Egbetade, G.O. and Oyedele, T.A., 2015. Effect of a school-based oral health education programme on use of recommended oral self-care for reducing the risk of caries by children in Nigeria. *International journal of paediatric dentistry*, *25*(4), pp.282-290.

Frencken, J.E., Borsum-Andersson, K., Makoni, F., Moyana, F., Mwashaenyi, S. and Mulder, J., 2001. Effectiveness of an oral health education programme in primary schools in Zimbabwe after 3.5 years. *Community Dentistry and Oral Epidemiology*, *29*(4), pp.253-259.

Frencken, J.E., de Amorim, R.G., Faber, J. and Leal, S.C., 2011. The Caries Assessment Spectrum and Treatment (CAST) index: rational and development. *International dental journal*, *61*(3), pp.117-123.

Ganesh AS, Bhat PK, Jyothi C. Initial impact of health education program on oral health, knowledge and awareness among 15 year old children of Government High school, Sarakki, Bangalore. J Ind Assoc Pub Health Dent 2007; 10: pp57–65

Gauba, A., Bal, I.S., Jain, A. and Mittal, H.C., 2013. School based oral health promotional intervention: Effect on knowledge, practices and clinical oral health related parameters.*Contemporary clinical dentistry*,*4*(4), pp.493.

Giacaman, R.A., Bustos, I.P., Bazán, P. and Mariño, R.J., 2018. Oral health disparities among adolescents from urban and rural communities of central Chile.

Graça, S.R., Albuquerque, T.S., Luis, H.S., Assunção, V.A., Malmqvist, S., Cuculescu, M., Slusanschi, O., Johannsen, G., Galuscan, A., Podariu, A.C. and Johannsen, A., 2019. Oral health knowledge, perceptions, and habits of adolescents from Portugal, Romania, and Sweden: A comparative study. *Journal of International Society of Preventive & Community Dentistry*, *9*(5), pp.470.

Hagberg, L. and Sjödahl, J., 2007. Knowledge and experience of oral health among secondary school students in Zambia

Haleem, A., Siddiqui, M.I. and Khan, A.A., 2012. School-based strategies for oral health education of adolescents-a cluster randomized controlled trial. *BMC oral health*, *12*(1), pp.54.

Hamoonga, A., Anthony, S.N. and Siziya, S., 2015. Knowledge, attitudes and practices on oral hygiene among 12 years old school children in Luanshya, Zambia. *Tanzania Dental Journal*, *19*(1), pp.5-10.

Haque, S.E., Rahman, M., Itsuko, K., Mutahara, M., Kayako, S., Tsutsumi, A., Islam, M.J. and Mostofa, M.G., 2016. Effect of a school-based oral health education in preventing untreated dental caries and increasing knowledge, attitude, and practices among adolescents in Bangladesh. *BMC Oral health*, *16*(1), pp.44.

Hilgert, L.A., Leal, S.C., Mulder, J., Creugers, N.H.J. and Frencken, J.E., 2015. Caries-preventive effect of supervised toothbrushing and sealants. *Journal of dental research*, *94*(9), pp.1218-1224.

Jamelli, S.R., Rodrigues, C.S., De Lira, P.I., 2010. Nutritional status and prevalence of dental caries among 12-year-old children at public schools: a case-control study. Oral Health Prev. Dent. 8, pp77–84.

Jepsen, S., Blanco, J., Buchalla, W., Carvalho, J.C., Dietrich, T., Dörfer, C., Eaton, K.A., Figuero, E., Frencken, J.E., Graziani, F. and Higham, S.M., 2017. Prevention and control of dental caries and periodontal diseases at individual and population level: consensus report of group 3 of joint EFP/ORCA workshop on the boundaries between caries and periodontal diseases. *Journal of clinical periodontology*, *44*, pp.S85-S93.

Kahabuka, F.K., Petersen, P.E. and Mbawalla, H.S., 2018. Adolescents' Health Behaviours In Relation to Dental and Medical Consultation in Tanzania. *International Journal of Health Sciences and Research*, *8*(2), pp.73-82.

Kikwilu, E.N., Frencken, J. and Mulder, J., 2009. Impact of Atraumatic Restorative Treatment (ART) on the treatment profile in pilot government dental clinics in Tanzania. *BMC oral health*, *9*(1), pp.14.

Kolawole, K.A., Oziegbe, E.O. and Bamise, C.T., 2011. Oral hygiene measures and the periodontal status of school children. *International journal of dental hygiene*, *9*(2), pp.143-148.

Krisdapong, S., Prasertsom, P., Rattanarangsima, K. and Sheiham, A., 2013. Sociodemographic differences in oral health-related quality of life related to dental caries in thai school children.*Community Dent Health*,*30* (2), pp.112-8.

Lage, C.F., Fulgencio, L.B., Corrêa-Faria, P., Serra-Negra, J.M., Paiva, S.M. and Pordeus, I.A., 2017. Association between dental caries experience and sense of coherence among adolescents and mothers. *International journal of paediatric dentistry*,*27*(5), pp.412-419.

Leal, S.C., Bronkhorst, E.M., Fan, M. and Frencken, J.E., 2012. Untreated cavitated dentine lesions: impact on children's quality of life.*Caries research*, *46*(2), pp.102-106.

Lee, J.Y., Watt, R.G., Williams, D.M. and Giannobile, W.V., 2017. A new definition for oral health: implications for clinical practice, policy, and research. 1. *Journal of Dental Reserch.*96(2), pp125-127.

Lian, C.W., Phing, T.S., Chat, C.S., Shin, B.C., Baharuddin, L.H. and Che'Jalil, Z.J., 2010. Oral health knowledge, attitude and practice among secondary school students in Kuching, Sarawak. *Archives of Orofacial Sciences*, *5*(1), pp.9-16.

Löe, H., 1967. The gingival index, the plaque index and the retention index systems. *The Journal of Periodontology*, *38*(6), pp.610-616.

Lyra, M.C.A., Cruz, M., Menezes, V. and Heimer, M.V., 2015. Association between sense of coherence and dental caries experience in adolescents. *Pesquisa Brasileira em Odontopediatria e Clínica Integrada*, *15*(1), pp.235-241.

Malele-Kolisa, Y., Yengopal, V., Igumbor, J., Nqcobo, C.B. and Ralephenya, T.R., 2019. Systematic review of factors influencing oral health-related quality of life in children in Africa. *African journal of primary health care & family medicine*,*11*(1), pp.1-12**.**

Mapengo, M.A.A., Marsicano, J.A., de Moura, P.G., Sales-Peres, A., Hobdell, M. and Sales-Peres, S.H.D.C., 2010. Dental caries in adolescents from public schools in Maputo, Mozambique. *International dental journal*, *60*(4), pp.273-281.

Marinho, V.C.C., 2009. Cochrane reviews of randomized trials of fluoride therapies for preventing dental caries. *European Archives of Paediatric Dentistry*, *10*(3), pp.183-191.

Mashoto, K.O., Åstrøm, A.N., David, J. and Masalu, J.R., 2009. Dental pain, oral impacts and perceived need for dental treatment in Tanzanian school students: a cross-sectional study. *Health and quality of life outcomes*, *7*(1), pp.73.

Mashoto, K.O., Åstrøm, A.N., David, J. and Masalu, J.R., 2009. Dental pain, oral impacts and perceived need for dental treatment in Tanzanian school students: a cross-sectional study. *Health and quality of life outcomes*, *7*(1), pp.73.

Mashoto, K.O., Astrom, A.N., Skeie, M.S. and Masalu, J.R., 2010. Socio-demographic disparity in oral health among the poor: a cross sectional study of early adolescents in Kilwa district, Tanzania. *BMC Oral health*, *10*(1), pp.7.

Mathur, M.R., Tsakos, G., Millett, C., Arora, M. and Watt, R., 2014. Socioeconomic inequalities in dental caries and their determinants in adolescents in New Delhi, India. *BMJ open*, *4*(12), pp.e006391.

Mbawalla, H.S., Masalu, J.R. and Åstrøm, A.N., 2010. Socio-demographic and behavioural correlates of oral hygiene status and oral health related quality of life, the Limpopo-Arusha school health project (LASH): a cross-sectional study. *BMC pediatrics*, *10* (1), pp.87.

Mbawalla, H.S., Mtaya, M., Masalu, J.R., Brudvik, P. and Astrom, A.N., 2011. Discriminative ability of the generic and condition-specific Child-Oral Impacts on Daily Performances (Child-OIDP) by the Limpopo-Arusha School Health (LASH) Project: A cross-sectional study. *BMC pediatrics*, *11*(1), pp.45.

McDade, T.W., Chyu, L., Duncan, G.J., Hoyt, L.T., Doane, L.D. and Adam, E.K., 2011. Adolescents' expectations for the future predict health behaviors in early adulthood. *Social science & medicine*, *73*(3), pp.391-398.

McKittrick TR., Jacobsen KH., 2014. Oral hygiene practices among middle-school students in 44 low-and middle-income countries. *International dental journal*. 64(3):pp164-70.

Melo, P., Fine, C., Malone, S., Frencken, J.E. and Horn, V., 2018. The effectiveness of the Brush Day and Night programme in improving children's toothbrushing knowledge and behaviour. *International dental journal*, *68*, pp.7-16.

Msyamboza KP, Phale E, Namalika JM, Mwase Y, Samonte GC, Kajirime D, Chalila PD, Potani R, Mwale GC, Kathyola D, Mukiwa W. Magnitude of dental caries, missing and filled teeth in Malawi: National Oral Health Survey. BMC Oral Health.2016*;* pp16:29

Mtaya, M., Åstrøm, A.N. and Tsakos, G., 2007. Applicability of an abbreviated version of the Child-OIDP inventory among primary schoolchildren in Tanzania. *Health and quality of life outcomes*, *5*(1), pp.40.

Mtaya, M., Brudvik, P. and Åstrøm, A.N., 2009. Prevalence of malocclusion and its relationship with socio-demographic factors, dental caries, and oral hygiene in 12-to 14-year-old Tanzanian schoolchildren. *The European Journal of Orthodontics*, *31*(5), pp.467-476.

Murakami, S., Mealey, B.L., Mariotti, A. and Chapple, I.L., 2018. Dental plaque–induced gingival conditions. *Journal of clinical periodontology*, *45*, pp.S17-S27.

Naidu, J. and Nandlal, B., 2017. Evaluation of the effectiveness of a primary preventive dental health education programme implemented through school teachers for primary school children in Mysore city. *Journal of International Society of Preventive & Community Dentistry*, *7*(2), pp.82.

Nurelhuda N. M ., Ahmed MF., Trovik TA., Astrom AN. 2010. Evaluation of oral health-related quality of life among Sudanese schoolchildren using Child-OIDP inventory. *Health Qual Life Outcomes*, *8*(1), pp.152.

Nurelhuda, N.M., Trovik, T.A., Ali, R.W. and Ahmed, M.F., 2009. Oral health status of 12-year-old school children in Khartoum state, the Sudan; a school-based survey. *BMC oral health*, *9*(1), pp.15.

Nyamuryekunge, K.K., 2012. *Health and Oral Health related knwoledge, attitudes and behaviors-a study of secondary school students in Dar es Salaam, Tanzania. A cross sectional study of 16-20 year old students* (Master's thesis, The University of Bergen).

Okemwa, K.A., Gatongi, P.M. and Rotich, J.K., 2010. The oral health knowledge and hygiene practices among primary school children aged 5-17 years in a rural area of Uasin Gishu District, Kenya. *Journal of Dentistry and Oral Hygiene*.

Okullo, I., Åstrøm, A.N. and Haugejorden, O., 2004. Social inequalities in oral health and in use of oral health care services among adolescents in Uganda.*International Journal of Paediatric Dentistry*, *14*(5), pp.326-335.

Oredugba, F.A., 2004. Oral health care knowledge and practices of a group of deaf adolescents in Lagos, Nigeria. *Journal of public health dentistry*, *64*(2), pp.118-120.

Oyedele, T.A., Fadeju, A.D., Adeyemo, Y.I., Nzomiwu, C.L. and Ladeji, A.M., 2018. Impact of oral hygiene and socio-demographic factors on dental caries in a suburban population in Nigeria. *European Archives of Paediatric Dentistry*, *19*(3), pp.155-161.

Pakpour, A.H., Gholami, M., Gellert, P., Yekaninejad, M.S., Dombrowski, S.U. and Webb, T.L., 2016. The effects of two planning interventions on the oral health behavior of Iranian adolescents: a cluster randomized controlled trial. *Annals of Behavioral Medicine*, *50*(3), pp.409-418.

Pakpour, A.H., Hidarnia, A., Hajizadeh, E., Kumar, S., Harrison, A., 2011. The status of dental caries and related factors in a sample of Iranian adolescents. Med. Oral Patol. Oral Cir. Bucal 16

Pattussi, M.P., Hardy, R. and Sheiham, A., 2006. The potential impact of neighborhood empowerment on dental caries among adolescents. *Community Dentistry and Oral Epidemiology*, *34*(5), pp.344-350.

Peres, K.G., Cascaes, A.M., Leão, A.T.T., Côrtes, M.I.D.S. and Vettore, M.V., 2013. Sociodemographic and clinical aspects of quality of life related to oral health in adolescents. *Revista de saude publica*, *47*, pp.19-28.

Petersen, P.E., 2004. Challenges to improvement of oral health in the 21st century—the approach of the WHO Global Oral Health Programme. *International dental journal*, *54*(S6), pp.329-343.

Petersen, P.E., 2004. Improvement of oral health in Africa in the 21st century-the role of the WHO Global Oral Health Programme.*African Journal of Oral Health*, *1*(1), pp.2-16.

Petersen, P.E., Hunsrisakhun, J., Thearmontree, A., Pithpornchaiyakul, S., Hintao, J., Jürgensen, N. and Ellwood, R.P., 2015. School-based intervention for improving the oral health of children in southern Thailand. *Community Dental Health*, *32*(1), pp.44-50.

Pitts, N.B., Zero, D.T., Marsh, P.D., Ekstrand, K., Weintraub, J.A., Ramos-Gomez, F., Tagami, J., Twetman, S., Tsakos, G. and Ismail, A., 2017. Dental caries. *Nature reviews Disease primers*, *3*(1), pp.1-16.

Qadri, G., Alkilzy, M., Franze, M., Hoffmann, W. and Splieth, C., 2018. School-based oral health education increases caries inequalities. *Community Dent Health*, *35*(3), pp.153-159.

Reddy, M.P., Lakshmi, S.V., Kulkarni, S., Doshi, D., Reddy, B.S. and Shaheen, S.S., 2016. Impact of oral health education on plaque scores with and without periodic reinforcement among 12-year-old school children. *Journal of Indian Association of Public Health Dentistry*, *14*(2), pp.116.

Ribeiro, A.P.D., Almeida, R.F., Medonca, J.G.A. and Leal, S.C., 2018. Oral health and its effect on the academic performance of children and adolescents. *Pediatric dentistry*, *40*(1), pp.12-17.

Roncalli, A.G., Sheiham, A., Tsakos, G. and Watt, R.G., 2015. Socially unequal improvements in dental caries levels in B razilian adolescents between 2003 and 2010. *Community dentistry and oral epidemiology*, *43*(4), pp.317-324.

Sanadhya, Y.K., Thakkar, J.P., Divakar, D.D., Pareek, S., Rathore, K., Yousuf, A., Ganta, S., Sobti, G., Maniar, R., Asawa, K. and Tak, M., 2014. Effectiveness of oral health education on knowledge, attitude, practices and oral hygiene status among 12–15-year-old schoolchildren of fishermen of Kutch district, Gujarat, India. *International maritime health*, *65*(3), pp.99-105.

Sharva, V., Reddy, V., Bhambal, A. and Agrawal, R., 2014. Prevalence of gingivitis among children of urban and rural areas of Bhopal district, India. *Journal of clinical and diagnostic research: JCDR*, *8*(11), pp.ZC52.

Shenoy, R.P. and Sequeira, P.S., 2010. Effectiveness of a school dental education program in improving oral health knowledge and oral hygiene practices and status of 12-to 13-year-old school children. *Indian journal of dental research*, *21*(2), pp.253.

Simangwa, L.D., Åstrøm, A.N., Johansson, A., Minja, I.K. and Johansson, A.K., 2018. Oral diseases and socio-demographic factors in adolescents living in Maasai population areas of Tanzania: a cross-sectional study. *BMC oral health*, *18*(1), pp. 200.

Simushi N, Nyerembe S A, Sasi R, Siziya S. . 2018. Dental caries on permanent dentition in primary school children — ndola, zambia, 2017 . *Health Press Zambia Bull* 2(4); pp 5-16.

Singh, S., 2011. Dental caries rates in South Africa: implications for oral health planning. *Southern African Journal of Epidemiology and Infection,26* (4), pp.259-261.

Slade, G.D., 1997. Measuring oral health and quality of life. *Chapel Hill*, *3*.

Smith, P.G., Morrow, R.H. and Ross, D.A., 2015. Types of intervention and their development. In *Field Trials of Health Interventions: A Toolbox. 3rd edition*. OUP Oxford.

Smits, J. and Steendijk, R., 2015. The international wealth index (IWI). *Social Indicators Research*, 122(1), pp.65-85.

Smyth, E., Caamaño, F. and Fernández-Riveiro, P., 2007. Oral health knowledge, attitudes and practice in 12-year-old schoolchildren. *Medicina Oral, Patología Oral y Cirugía Bucal (Internet)*, *12*(8), pp.614-620.

Stein, C., Santos, N.M.L., Hilgert, J.B. and Hugo, F.N., 2018. Effectiveness of oral health education on oral hygiene and dental caries in schoolchildren: Systematic review and meta-analysis. *Community dentistry and oral epidemiology*, *46*(1), pp.30-37.

Sukhabogi, J.R., Shekar, C.B.R., Hameed, I.A., Ramana, I.V. and Sandhu, G., 2014. Oral health status among 12 and 15 year old children from government and private schools in Hyderabad, Andhra Pradesh, India. *Annals of medical and health sciences research*, *4*(3), pp.272-277.

Trombelli, L., Farina, R., Silva, C.O. and Tatakis, D.N., 2018. Plaque-induced gingivitis: Case definition and diagnostic considerations. *Journal of clinical periodontology*, *45*, pp.S44-S67.

World Health Organization and Centers for Disease Control and Prevention (CDC, 2013. Global school-based student health survey (GSHS).

Usha, G.V., Thippeswamy, H.M. and Nagesh, L., 2013. Comparative assessment of validity and reliability of the Oral Impacts on Daily Performance (OIDP) frequency scale: a cross-sectional survey among adolescents in Davanagere city, Karnataka, India. *International journal of dental hygiene*, *11*(1), pp.28-34.

Vadiakas, G., Oulis, C.J., Tsinidou, K., Mamai-Homata, E. and Polychronopoulou, A., 2012. Oral hygiene and periodontal status of 12 and 15-year-old Greek adolescents. A national pathfinder survey.*European Archives of Paediatric Dentistry*,*13*(1), pp.11-20.

Van der Walt, M., Van Wyk, P.J., Bester, J.K. and Becker, P.J., 2018. The effectiveness of a tooth brushing programme for children in the Ehlanzeni district of Mpumalanga. *South African Dental Journal*, *73*(10), pp.604-611.

Varenne, B., Petersen, P.E. and Ouattara, S., 2006. Oral health behaviour of children and adults in urban and rural areas of Burkina Faso, Africa. *International dental journal*, *56*(2), pp.61-70.

Veiga, N.J., Pereira, C.M., Ferreira, P.C. and Correia, I.J., 2015. Prevalence of dental caries and fissure sealants in a Portuguese sample of adolescents. *PloS one*, *10* (3).pp 1-12

Vernazza, C.R., Rolland, S.L., Chadwick, B. and Pitts, N., 2016. Caries experience, the caries burden and associated factors in children in England, Wales and Northern Ireland 2013.*British dental journal*,*221*(6), pp.315.

Wickremasinghe WM and Ekanayake 2017. Effectiveness of a health education intervention based on the Health Belief Model to improve oral health behaviours among adolescents. *Asian Pacific Journal of Health Scinces*.4 (1) pp 48-55.

World Health Organization, 2001.The second decade: improving adolescent health and development (No. WHO/FRH/ADH/98.18 Rev. 1). Geneva: World Health Organization.

Yang, Y.H., Sue, R.L., Warnakulasuriya, S. and Dasanayake, A.P., 2009. Promoting better oral health practices among aboriginal Taiwanese adolescents: a school based oral health education intervention program. *Journal of health care for the poor and underserved*, *20*(4), pp.41-50.

Yazdani, R., Vehkalahti, M.M., Nouri, M. and Murtomaa, H., 2009. School-based education to improve oral cleanliness and gingival health in adolescents in Tehran, Iran. *International Journal of Paediatric Dentistry*, *19*(4), pp.274-281.

Yekaninejad, M.S., Eshraghian, M.R., Nourijelyani, K., Mohammad, K., Foroushani, A.R., Zayeri, F., Pakpour, A.H., Moscowchi, A. and Tarashi, M., 2012. Effect of a school-based oral health-education program on I ranian children: results from a group randomized trial. *European journal of oral sciences*, *120* (5), pp.429-437.

Zhu, L., Petersen, P.E., Wang, H.Y., Bian, J.Y. and Zhang, B.X., 2003. Oral health knowledge, attitudes and behaviour of children and adolescents in China. *International dental journal*, *53*(5), pp.289-298

8.0 Appendices

Appendix 1 Adolescents ‘English Questionnaire

Adolescents ID No

EFECTIVENESS OF MODIFIED BASIC PACKAGE OF ORAL CARE ON ORAL HEALTH STATUS AND RELATED QUALITY OF LIFE AMONG ZAMBIAN ADOLESCENTS-: A FIELD TRIAL

Instructions

This questionnaire has a three (3) parts with a total of thirty-nine (39) questions. Answer all questions by ticking in inside the box corresponding to answer of your choice. Some questions may have more than one answer.

Part 1 Demographic information of adolescent and parents/ guardians

1. What is your sex?
   1 Male ☐
   2 Female ☐

2. What is your birth date?
   ☐☐ ☐☐ ☐☐
   DD MM YY

3. Where do you live?
   Ward …………………………………..
   Disrtict …………………………………..

4 Do you live with both parents at home?
   1. Yes ☐
   2. No, Mother only ☐
   3. No, Father only ☐
   4. No, I live with guardian ☐
   5. Others Specify…………………...

5 What is the highest education level of your father?
   1. No formal education ☐
   2. Primary education ☐
   3. Secondary education ☐
   4. College/University ☐
   5. I don’t know ☐
   6. Not applicable ☐

6 What is the highest education level of your mother?

1. No formal education ☐
2. Primary education ☐
3. Secondary education ☐
4. College/University ☐
5. I don't know ☐
6. Not applicable ☐

7 How many members make your family (including your parents /guardians, yourself, your brothers and sisters and other people permanently living at your home (excluding visitors) ……..

8 How many children are living and taken care of by your parents/ guardians (including yourself, your brothers and sisters and other children 19 years or younger (excluding visitors) living at your home ……..

9 Do you have the following at your house? (You can tick more than 1)

	Items	Yes	No
1	Television	☐	☐
2.	Refrigerator	☐	☐
3.	Phone	☐	☐
4.	Car	☐	☐
5	Motorcycle	☐	☐
5.	Bicycle	☐	☐

10 How many sleeping rooms do the house you live in has?

	Items	
1	Zero to One	☐
2.	Two	☐
3	Three or more	☐

11 Which of the following describe the floor of your house?

	Items	
1	None , mud or dung	☐
2.	Cemented/ concrete/ raw wood	☐
3.	Finished floor with carpet , tiles or ceramic	☐

12 Do you have any of the following at home?

		Yes	No
1.	Dish washer		
2	Dish dryer		
3.	Computer		
4.	Motor boat		
5.	Air conditioner		
6	Generator		

13 Which of the following describe a toilet at your home?

	Items	
1	Traditional pit latrine toilet/no toilet	
2.	Public toilet/Improved pit latrine	
3	Private Flash toilet	

14 Where do you get water at your home?

	Items	
1	Unprotected well, bore hole shallow well surface water	
2.	Public tap, protected well, tanker track	
3	Bottled or pipe water in dwelling or premises	

15 Does your house have electricity?

1. No
2. Yes

Part 2 Adolescents oral health knowledge and behaviors

16. I can avoid tooth decay by accomplishing the following;

		1=yes	2=no
1.	Restricting eating of sugar containing foods such as vitumbua, filters, cake, chocolate to less than five times per day	☐	☐
2	Restricting drinking of sugar sweetened drinks such as tea, coffee, porridge, coke, fanta, mukoyo or juice to less than five times per day	☐	☐
3.	Using fluoride toothpaste to brush my teeth at least twice a day spitting the foam but not rinsing it out	☐	☐
4	Visiting a dentist at least once a year for dental check up	☐	☐

17. I can avoid bleeding gums by fulfilling the following;

		1=yes	2=no
1.	Restricting eating/drinking of sugar containing foods/ drinksto less than five times per day	☐	☐
2.	Brushing teeth for 2 minutes at least twice a day	☐	☐
3.	Visiting a dentist at least once a year for dental check up	☐	☐
4	Using a dental floss to clean between my teeth	☐	☐
5	Using a tooth pick to clean between my teeth	☐	☐

18. When should a tooth brush be changed

		1=yes	2=no
1.	When the brush head is broken	☐	☐
2.	Once a month	☐	☐
3.	Three months or when bristlesflare out (bend)	☐	☐
4	I don't know	☐	☐

19. During the past 30 days how often did you drink sugar sweetened drinks such tea, coffee, porridge, coke, fanta, mukoyo or juice?

1.	I didn't	☐
2.	Occasionally per week	☐
3.	Once per day	☐
4.	Twice to four times per day	☐
5.	Five times or more per day	☐

20. During the past 30 days how often did you eat sugar containing foods such vitumbua, filters, cake, chocolate?

1. I didn't
2. Occasionally per week
3. Once per day
4. Twice to four times per day
5. Five times or more per day

21. How many times in a day did you brush your teeth in the past 30 days?

1. I didn't brush at all
2. I brushed but not everyday
3. I brushed once a day
4. I brushed twice or more a day

22. What do you use to clean your teeth?

1. Nothing
2. Finger
3. Chewing stick
4. Plastic tooth brush

23. If you use a plastic tooth brush how often do you change it?

1. After 1 month
2. After 2 months
3. After 3 months
4 When worn out (bristles bent)

24. Do you use toothpastes for tooth brushing?

1. No
2. Yes

25. If you do, does the tooth paste contain fluoride

1. No
2. Yes
3 I don't know

26. How often did you use fluoridated toothpaste during the past 30 days?

1. I didn't use tooth paste
2. I used but not everyday
3. I used once a day
4. I used twice a day or more

27. How many times did you attend for dental checkup in past 1 year?

1. I did not attend ☐
2. I attended once ☐
3. I attended twice or more ☐

28. If you did not attend for check up, what is the reason?

1. I did not have any problem ☐
2. My parents didn't have money ☐
3. My parents didn't have time ☐
4. The clinic is very far ☐
5. I had fear of a dentist ☐
6. I used traditional medicine ☐
7 Others Specify ……………………….

29. Did you experience any of the following dental problems in the past 3 months?

		1=yes	2=no
1.	Painful tooth	☐	☐
2.	Painful gums	☐	☐
3.	Swollen gums	☐	☐
4	Bleeding gums	☐	☐
5.	Injury to teeth	☐	☐
6.	Bad teeth arrangement	☐	☐
7.	Others Specify ……………………..		

30. How would you describe the health of your teeth?

1. Very poor ☐
2. Poor ☐
3. Good ☐
4. Very good ☐

31. How would you describe the health of your gums?

1. Very poor ☐
2. Poor ☐
3. Good ☐
4. Very good ☐

Part 3: Impact of Oral diseases on Adolescents daily performance

32. During the past three months how often the problems with your mouth and teeth caused you any difficulty with eating or enjoying food?
    1. Never ☐
    2. Once or twice a month ☐
    3. Once or twice a week ☐
    4. Every or nearly everyday ☐

33. During the past three months how often the problems with your mouth and teeth caused you any difficulty with speaking or pronouncing words?
    1. Never ☐
    2. Once or twice a month ☐
    3. Once or twice a week ☐
    4. Every or nearly everyday ☐

34. During the past three months how often the problems with your mouth and teeth caused you any difficulty with cleaning teeth?
    1. Never ☐
    2. Once or twice a month ☐
    3. Once or twice a week ☐
    4. Every or nearly everyday ☐

35. During the past three months how often the problems with your mouth and teeth caused you any difficulty with sleeping and relaxing?
    1. Never ☐
    2. Once or twice a month ☐
    3. Once or twice a week ☐
    4. Every or nearly everyday ☐

36. During the past three months how often the problems with your mouth and teeth caused you any difficulty with smiling, laughing, showing teeth without embarrassment?
    1. Never ☐
    2. Once or twice a month ☐
    3. Once or twice a week ☐
    4. Every or nearly everyday ☐

37. During the past three months how often the problems with your mouth and teeth caused you any difficulty in maintaining usual emotional state without being irritable?

1. Never ☐
2. Once or twice a month ☐
3. Once or twice a week ☐
4. Every or nearly everyday ☐

38. During the past three months how often the problems with your mouth and teeth caused you any difficulty with carrying out school activities such as attending class or social role?

1. Never ☐
2. Once or twice a month ☐
3. Once or twice a week ☐
4. Every or nearly everyday ☐

39. During the past three months how often the problems with your mouth and teeth caused you any difficulty with enjoying contact with people?

1. Never ☐
2. Once or twice a month ☐
3. Once or twice a week ☐
4. Every or nearly everyday ☐

THANK YOU

Appendix 2: Clinical data collection form

EFECTIVENESS OF MODIFIED BASIC PACKAGE OF ORAL CARE ON ORAL HEALTH STATUS AND RELATED QUALITY OF LIFE AMONG ZAMBIAN ADOLESCENTS-:A FIELD TRIAL

Adolescent's Id	☐☐☐☐	Gender	☐	(1) Boy<br>(2) Girl		Birth date?<br>☐☐ DD ☐☐ MM ☐☐ YY
Examination date?<br>☐☐ DD ☐☐ MM ☐☐ YY		Data<br>(1) Baseline<br>(2) follow up (circle)<br>1st 2nd 3rd 4th			Duplicate ☐<br>(1) Original<br>(2) Duplicate	Examiner ID<br>☐

**Part 1 : Caries Assessment Spectrum and Treatment (CAST) record form**														
P **Surface**	**Tooth Number**													
	17	16	15	14	13	12	11	21	22	23	24	25	26	27
D														
O														
M														
B														
L														
	47	46	45	44	43	42	41	31	32	33	34	35	36	37
D														
O														
M														
B														
P L														

art 1 key CAST Codes

Characteristic	Code	Description
Sound	0	No visible evidence of a distinct carious lesion is present
Sealant	1	Pits and/ or fissure are at least partially covered with a sealant material
Restoration	2	A cavity is restored with an (in)direct restorative material
Enamel	3	Distinct visual change in enamel only; a clear caries-related discolouration is visible, with or without localised enamel breakdown
Dentine	4	Internal caries-related discolouration in dentine; the discoloured dentine is visible through the enamel, which may or may not exhibit a visible localised breakdown
	5	Distinct cavitation into dentine; the pulp chamber is intact
Pulp	6	Involvement of the pulp chamber; distinct cavitation reaching the pulp chamber, or only root fragments are present
Abscess/ fistula	7	A pus-containing swelling or a pus-releasing sinus tract related to a tooth with pulpal involvement
Lost	8	The tooth has been removed because of dental caries
Other	9	Does not match with any of the other descriptions

Adolescents Id No………..

**Part 2 : Plaque Index by Silness &Löe**			
	**Index teeth**		
Surface	16	11	26
Buccal			
Lingual			
	46	41	36
Buccal			
Lingual			

Key for part 2

B=Buccal, L=Lingual, M=Mesial, D= Distal , PI=Plaque Index (Total score for B,L, M and D divide by 4)

Codes

0	Plaque not visible nor cannot be wiped off with the explorer
1	Plaque not visible but can be wiped off with the explorer
2	Plaque is visible along the gingival no need to probe
3	Thick plaque is visible along gingival margin

Notes

- If the index tooth is missing score the nearest tooth in that sextant
- If there is no tooth in that sextant indicate X
- The overall score is sum of the 12 surfaces scores (maximum of 36)

Adolescents Id No………..

**Part 3: Gingival bleeding scores according to Silness-Löe plaque index**			
	**Index teeth**		
**Surface**	16	11	26
Buccal			
Lingual			
	46	41	36
Buccal			
Lingual			

Codes 0= No inflammation (Normal gingival, no discoloration, no bleeding)

1= Mild inflammation (slight color change, mild alteration of gingival surface, no bleeding)

2= Moderate inflammation (erythema, swelling, bleeding on probing or when pressure is applied)

3= Severe inflammation (severe erythema and swelling and tendency towards spontaneous bleeding, some ulceration)

Appendix 3 -CONSENT FORM (English version)

Study Title: **EFECTIVENESS OF MODIFIED BASIC PACKAGE OF ORAL CARE ON ORAL HEALTH STATUS AND RELATED QUALITY OF LIFE AMONG ZAMBIAN ADOLESCENTS-: A FIELD TRIAL**

Introduction

Hello, My name is Dr. Severine Nyerembe Anthony (Senior Lecturer at Michael Chilufya Sata, School of Medicine, Copperbelt University and PhD student). We are carrying out a research on oral health knowledge and behaviour; oral health status and its effects on quality of life among adolescents in Copperbelt province, Zambia

Purpose of the study

The study will assess the success of World Health Organization (WHO) basic package of oral care which include provision of emergence treatment such as tooth extraction, distribution of toothpastes, filling of teeth and giving oral health education in improving oral health related behaviors, oral health and quality of life among adolescents in Copperbelt province, Zambia

Study procedures

We will ask about your child's age, sex, parental education status, family living conditions, oral health knowledge and behaviors, and effects of oral diseases on his/her daily performance. We will also check your child's teeth and gums. **A school at which your child attends will be allocated by chance to either receive treatment or continue with self-standard of oral care. The pupils at schools allocated to treatment group will receive basic package of oral care explained on study purpose above. The pupils from schools allocated to self -standard of care will be encouraged to continue with their normal standard of oral care, however if anyone in this group will be found with oral illness during baseline data collection will be referred to nearest health facility for treatment**. Participants requiring field treatment (e. g fillings without drilling and simple extraction) will receive treatment at school but those requiring further investigations and management will be referred to nearby health facilities.

Confidentiality

All information that will be collected from your child and oral health examination results will be kept confidential and will only be used for research purposes.

Study Benefits

Taking part in this study will enable you to get feedback on the status of oral health (health of the gums and teeth) of your child at the beginning and at the end of the study. Your child's information and others participating in the study will collectively be used by policy makers in addressing adolescents' oral health problems in Zambia.

Study Risks

We do not expect that any harm will happen to your child because of participating in this study. All treatment and follow up care will be done according to Zambia dental standard treatment guidelines by qualified registered dental personnel.

Voluntariness

Your child's participation in this study is completely voluntary. Parental/guardians refusal to participate or withdrawal from the study will not involve penalty or loss of any benefits to which a child is otherwise entitled. If you choose for your child not to participate in the study or decide to stop him/her from participating at any time he/she will continue to be treated normally. If for any reason you would wish him/her to come back into the study after withdrawal, we will be ready to accept him/her to continue with the study.

If you have any doubts or you wish to seek clarification on research please feel free to contact the main researcher on the address below:

Name: Dr Severine Nyerembe Anthony
Organisation: Copperbelt University, Michael Chilufya Sata School of Medicine.
Address: P.o Box 71191, Hillcrest, **Ndola**
E mail anthonyerembe1975@yahoo.com
Tel: +260966307828

If you have any complaints about the study, please contact the Secretary of the TDRC Ethics Review Committee at the following address:
The Secretary
TDRC Ethics Review Committee
Box 71769
Ndola
E mail: tdrc-ethics@tdrc.org.zm
Tel: +260 212 615444

I understand the information given to me and that my child's participation is completely voluntary and its purpose has been fully explained to me. I also understand that my child's rights and privacy will be respected.
Name of the parent/ guardian ……………………………………………

Signature or thumb print of parent/guardian……………………………………………

Name and Signature of person obtaining consent ……………………………………………

Date …………….. ………

Appendix 4- CONSENT FORM (Bemba version)

INSAMBU SHAKUSENDAMO ULUBALI MUKUFWAILISHA KWA FITUNGILILA NOKU CHINCHISHA UBUMI BWA MENO NE FIPONSHI MUKANWA, NO MULINGANYA PAKUFIKILISHYA UBUMI BWA MENO NE FIPONSHI MUKANWA KUMISEPELA MUCHALO CHA ZAMBIA.

Intashi

Mukwai, Ishina lyandi nine Dr. Severine Nyerembe Anthony (Ndipamasambililo yakalamba per sukulu likalamba ilya masambililo ya bumi iitwa Muhimbili University of Health and Allied Sciences, ku chalo chaku Tanzania. Kabili nine kafundisha mukalamba per sukulu likalamba ilya bumi, iitwa Michael Chilufya Sata, School of Medicine, Copperbelt University, mu Zambia.

Mukwa, ndipa mulimo waku fwailisha ifitungilila noku chinchisha ubumi mwa mukanwa, no mulinganya pakufikilishya ubumi bwa mukanwa kumisepela muchalo cha Zambia.

Ichotwimininepo pa kufwailisha kwesu

Ichikalamba chakutila tufwailikishe ifitungilila ubutantiko bwa bundapo bwa bumi bwa mukanwa no kutungilila ubumi bwesu muku pwisha ifilenga ukukana kwata umulinganya pakusanga ubumi bwa mukanwa mumisepela ba mu chalo cha Zambia.

Abalingile ukusendako ulubali mukufwailisha uku

Nga mulesuminisha ukuti umusepela wenu asendeko ulubali mukufwailisha uku, tulemilomba uku musuminisha ukwasuka amepusho ayo tuleipusha mukufwailisha kwesu, kabili tulelomba ukuti mwinga musuminisha ukuti to muchenchente mukanwa pakuti pakuti tumone nga muliko ubwafya bwa meno nangula ifiponshi pakuti tu lange ifya kuichingilila ku malwele aya meno ne fiponshi. Balasangwa ukuti balingile ubundapo ubuchililepabo twalabapela ili tuli munchito yaku fwailisha, tuli nokuba twala kwi sukulu likalamba ilya bumi pantu ekuli nechipatala chamalwele yamukanwa aya meno nefiposhi. Elyo abakwete amalwele ya mukanwa ayachililepo, kuti twabatuma kuchipatala chikalamba icha meno nefi poshi.

Munkama

Fyonse ifyalalandwa no kuchitwa kumwana wenu fyalaba ifya munkama, fyaku bomfyafye muku tungilila imilimo yakufwailisha kwesufye. Tapali nangula umo uutakumine kumilimo yakufwailisha uwingakwata ukwishiba pafyo twalanshanya no mwana wenu. Tatwakulalemba amashina pamapepala yamepusho ya pakufwailisha.

Ubusuma bwakusendamo ulubali mukufwailisha kwesu

Ubusuma bwakusuminisha umwana wenu ukusendamo ulubali muku fwailishakwesu bwakuti, umwana wenu ala yafwilishiwa ukukwata imibele iisuma iya ku tungilila ubumi busuma ubwa meno ne fiponshi, no chefyanyako amalwele ya meno no bubi fyakwata pabumi bwa munthu.

Ufyo twalasanga mukufwailisha kwesu ukupitila mumwana wenu nababmbi abalesendako ulubali mukufwailishakwesu, kuti fya afwa uku wamya nangula ukupanga amafunde yachalo yakuma muku pakamisha ubumi bwa mukanwa (ameno ne fiponshi) ubwa misepela ya muchal cha Mu Zambia. Kabili tukamweba nefikatundukamo mukufwailisha kwesu.

Ububi bwakusendako ulubali mukufwailisha

Tatulechetekela ukusangamo ububi nangubumo mukusuminisha umwana wenu ukusendamo ulubali mukufwailisha kwesu. Bonse ubundapo bwamalwele ya meno ne fiponshi bwalachitwa ukukonka no butantiko bwa chalo cha Zambia, kabili kundapwa no mubofi uwasuminishiwa na mafunde ya muchalo cha Zambia ukundapa abantu mufipatala.

Nsambu shakukana konkanyapo no kukonkanyapo

Chilifye kuli imwe uku salapo ukusuminisha nangula ukukanya umwana wenu uku sendako ulubali mukufwailisha kwesu. Kabili namukwata nsambu shakukanya umwana wenu ukukonkanyapo no bufwailisho bwesu, nangulafye mwachimusuminisha pakwamba. Kabili nga mulekabila umwanawenu ukwisa konkanyapo no bufwailisho tulechita, panuma yakumulesha, ifwe kwisa mupokelelafye chikulu namusuminisha ukukonkanyapo. Tapali umulandu nangu ubupusanu bwalabapo pa kukanya umwana wenu ukusendako ulubali mukufwailisha kwesu. Takuli amalipilo pakusendamo ulubali mukufwailisha kwesu.

Abakwipushako

Nganamukwata amepusho pakufwailisha kwesu uku, kuti mwaipusha ba kafwailisha mukalamba Ba Shin'ganga kabili bakafundisha pesukulu likalamba

Name: Dr Severine Nyerembe Anthony
Organisation: Copperbelt University, Michael Chilufya Sata School of Medicine.
Address: P.o Box 71191, Hillcrest, **Ndola**
E mail anthonyerembe1975@yahoo.com
Tel: +260966307828

Nganamukwatapo ifyo tamuleumfwikisha pensambu shenu pakusendapo ulubali mukufwailishakwesu kuti mwa tumina ba

The Secretary

TDRC Ethics Review Committee
Box 71769
Ndola
E mail: tdrc-ethics@tdrc.org.zm
Tel: +260 212 615444

Bushe mulesuminisha ukutila umwana wenu asendepo ulubali mukufwailisha kwesu?

nimbelenga muchipepala ichi. Na mepusho yonse nachikwata nayasukwa. Na sumina ukutila umwana wandi asendeko ulubali mukufwailisha uku.

Name of the parent/ guardian ………………………………………………
Signature or thumb print of parent/guardian……………………………………………
Name and Signature of person obtaining consent ……………………………………………
Date …………….. ………

Appendix 5 –ASSENT FORM (English version)

EFECTIVENESS OF MODIFIED BASIC PACKAGE OF ORAL CARE ON ORAL HEALTH STATUS AND RELATED QUALITY OF LIFE AMONG ZAMBIAN ADOLESCENTS-: A FIELD TRIAL

Assent to participate in the study

Introduction

Hello, my name is Dr. Severine Nyerembe Anthony (Senior Lecturer at Michael Chilufya Sata, School of Medicine, Copperbelt University and PhD student). We are carrying out a research on oral health knowledge and behaviour; oral health status and its effects on quality of life among adolescents in Copperbelt province, Zambia

Purpose of the study

The study will assess the success of World Health Organization (WHO) basic package of oral care which include provision of emergence treatment such as tooth extraction, distribution of toothpastes, filling of teeth and giving oral health education in improving oral health related behaviors, oral health and quality of life among adolescents in Copperbelt province, Zambia

Study procedures

We will ask questions on your age, sex, parental education status, family living conditions, oral health knowledge and behaviors, and effects of oral diseases on his/her daily performance. We will also check your teeth and gums. Your will either be allocated to observation group or intervention/ treatment group. The allocation will be by chance and those who will allocate to treatment will receive basic package of oral care explained on study purpose above. The observation group will be encouraged to continue with their normal standard of oral care however if anyone in this group will be found with oral illness during baseline data collection will be referred to nearest health facility for treatment. Participants requiring field treatment (e. g fillings without drilling and simple extraction) will receive treatment at school but those requiring further investigations and management will be referred to nearby health facilities.

Confidentiality

All information that will be collected from you and oral health examination results will be kept confidential and will only be used for research purposes.

Study Benefits

Taking part in this study will contribute towards improving your oral health behaviors; reduce oral health problems and their impacts on his/her daily performance. Your information and others participating in the study will collectively be used by policy makers in addressing

adolescents' oral health problems in Zambia. Furthermore, you will receive feedback of your oral health status at the beginning and upon completion of this study.

Study Risks

We do not expect that any harm will happen to you because of participating in this study. All treatment and follow up care will be done according to Zambia dental standard treatment guidelines by qualified registered dental personnel.

Voluntariness

Your participation in this study is completely voluntary. Refusal to participate or withdrawal from the study will not involve penalty or loss of any benefits to which you are otherwise entitled. If you choose not to participate in the study or decide to stop participating at any time you will continue to be treated normally. If for any reason you would wish to come back into the study after withdrawal, we will be ready to accept you to back.

If you have any doubts or you wish to seek clarification on research, please feel free to contact the main researcher on the address below:

Name: Dr Severine Nyerembe Anthony
Organization: Copperbelt University, Michael Chilufya Sata School of Medicine.
Address: P.o Box 71191, Hillcrest, **Ndola**
E mail anthonyerembe1975@yahoo.com
Tel: +260966307828

If you have any complaints about the study, please advise you parent/ guardian to contact the Secretary of the TDRC Ethics Review Committee at the following address:
The Secretary
TDRC Ethics Review Committee
Box 71769
Ndola
E mail: tdrc-ethics@tdrc.org.zm
Tel: +260 212 615444

I understand the information given to me and that my participation is completely voluntary and its purpose has been fully explained to me. I also understand that my rights and privacy will be respected.
Name of the participant …………………………………………………
Signature or thumb print of the participant ………………………………………………
Name and Signature of person obtaining assent ………………………………………………
Date …………….. ………

Appendix 6- Analysis Plan by objectives

No	Objective	Analysis	To be reported as
1	To determine prevalence and distribution of dental caries, periodontal conditions and oral impacts on daily performance among adolescents in Copperbelt Province, Zambia	Frequency distribution, chi-square test, logistic regression	Number and percentage, odds ratio with 95% confidence interval
2	To determine the association between dental caries and oral health related quality of life among adolescents in Copperbelt Province	Frequency distribution, chi-square test, hierarchical logistic regression	Number and percentage, odds ratio with 95% confidence interval
3	To assess the effects of a six months modified basic package of oral care intervention on knowledge and behaviors related to dental caries after 18 and 24 months of follow up.	Frequency distribution, chi-square test, generalized estimating estimation	Number and percentage, odds ratio with 95% confidence interval
4	To assess the effects of a six months modified basic package of oral care intervention on prevalence of dental caries after 6 and 12 months of follow up	Frequency distribution, chi-square test, generalized estimating estimation	Number and percentage, odds ratio with 95% confidence interval
5	To assess the effects of a six months modified basic package of oral care intervention on prevalence of oral impacts on daily performance after 18 and 24 months of follow up	Frequency distribution, chi-square test, generalized estimating estimation	Number and percentage, odds ratio with 95% confidence interval
