# Supplementary material for: The effect of the modified basic package of oral care on adolescent dental caries status in Zambia; a cluster randomized trial
Source: Front Oral Health. 2025 May 7;6:1542337. doi: 10.3389/froh.2025.1542337 (PMC12092211; doi:10.3389/froh.2025.1542337)
Supplement: Supplementary file 3 [file Datasheet3.pdf]

| Group | Study period | January 2021 to March 2023 | | | | | | | | | | | | | | | | | | | | | | | | | | |
|---|---|---|---|---|---|---|---|---|---|---|---|---|---|---|---|---|---|---|---|---|---|---|---|---|---|---|---|---|
| **Intervention & control** | **Year** | **2021** | | | | | | | | | | | | **2022** | | | | | | | | | | | | **2023** | | |
| | **Month** | **1** | **2** | **3** | **4** | **5** | **6** | **7** | **8** | **9** | **10** | **11** | **12** | **1** | **2** | **3** | **4** | **5** | **6** | **7** | **8** | **9** | **10** | **11** | **12** | **1** | **2** | **3** |
| | **Time points** | $t_1$ | | | | | $t_2$ | | | | | | $t_3$ | | | | | | $t_4$ | | $t_5$ | | | | | | $t_6$ | |
| | **Enrolment** | | | | | | | | | | | | | | | | | | | | | | | | | | | |
| | Permissions | | | | | | | | | | | | | | | | | | | | | | | | | | | |
| | Parental consent | | | | | | | | | | | | | | | | | | | | | | | | | | | |
| | Eligibility | | | | | | | | | | | | | | | | | | | | | | | | | | | |
| | Adolescent assent | | | | | | | | | | | | | | | | | | | | | | | | | | | |
| | **Baseline data** | | | | | | | | | | | | | | | | | | | | | | | | | | | |
| | Questionnaire | | | | | | | | | | | | | | | | | | | | | | | | | | | |
| | Examination | | | | | | | | | | | | | | | | | | | | | | | | | | | |
| | Allocation | | | | | | | | | | | | | | | | | | | | | | | | | | | |
| **Intervention** | **BPOC intervention** | | | | | | | | | | | | | | | | | | | | | | | | | | | |
| | OUT | | | | | | | | | | | | | | | | | | | | | | | | | | | |
| | ART | | | | | | | | | | | | | | | | | | | | | | | | | | | |
| | AFT | | | | | | | | | | | | | | | | | | | | | | | | | | | |
| | Peer-led OHE | | | | | | | | | | | | | | | | | | | | | | | | | | | |
| **Control** | **Routine oral care** | | | | | | | | | | | | | | | | | | | | | | | | | | | |
| **Intervention & control** | **Follow ups** | | | | | | | | | | | | | | | | | | | | | | | | | | | |
| | 1st follow up | | | | | | | | | | | | | | | | | | | | | | | | | | | |
| | 2nd follow up | | | | | | | | | | | | | | | | | | | | | | | | | | | |
| | **End of study** | | | | | | | | | | | | | | | | | | | | | | | | | | | |
| | Peer-led OHE | | | | | | | | | | | | | | | | | | | | | | | | | | | |

**Supplementary material 2 Schedule of study activities.**

**Time points summary:** $t_1$ =enrollment and baseline data collection (5months), $t_2$ =intervention (months), $t_3$ =no field activity (6 months), t4 = first follow up (2 months), $t_5$ = no field activity (6 months), $t_6$= second follow up (2 months)
