# Supplementary material for: The effect of the modified basic package of oral care on adolescent dental caries status in Zambia; a cluster randomized trial
Source: Front Oral Health. 2025 May 7;6:1542337. doi: 10.3389/froh.2025.1542337 (PMC12092211; doi:10.3389/froh.2025.1542337)
Supplement: Supplementary file 4 [file Datasheet4.pdf]

TROPICAL DISEASES RESEARCH CENTRE
Tel/Fax +260212 615444
tdrc-ethics@tdrc.org.zm
P O Box 71769
Ndola,
ZAMBIA

TDRC ETHICS REVIEW COMMITTEE
IRB REGISTRATION NUMBER : 00002911
FWA NUMBER : 00003729

TRC/C4/10/2020

23th October 2020

Dr. Severine Nyerembe Anthony
Copperbelt University
NDOLA

Dear Dr. Nyerembe,

RE: ETHICAL APPROVAL OF STUDY PROTOCOL

Reference is made to the protocol entitled **"Effectiveness of Modified basic package of Oral care on Oral Health status and related quality of life among Zambian adolescents: A field trial."**

On behalf of the Chairperson of the TDRC Ethics Review Committee (ERC), I wish to inform you that the Committee during its meeting held on 14th October 2020 in the TDRC Boardroom, reviewed your protocol and granted ethical approval.

You are further required to submit progress reports to the TDRC ERC twice a year.

Should there be any protocol modifications or amendments, you are required to notify the ERC and submit protocol amendments for approval.

You are now required to submit your protocol to the National Health Research Authority for final approval following the link: https://www.nhra.org.zm. A final report of the study should be submitted to the Ethics Review Committee Secretariat at the end of the study.

This approval is valid for the period 23th October 2020 to 23th October 2021

The Committee wishes you success in the execution of the study.

Yours faithfully,
TROPICAL DISEASES RESEARCH CENTRE

Sydney Mwanza
DEPUTY SECRETARY – TDRC Ethics Review Committee

TROPICAL DISEASES RESEARCH CENTRE
ETHICS REVIEW COMMITTEE
23 OCT 2020
P.O. BOX 71769
NDOLA, ZAMBIA

TROPICAL DISEASES RESEARCH CENTRE
Tel/Fax +260212 615444
P O Box 71769
tdrc-ethics@tdrc.org.zm
NDOLA, ZAMBIA

TDRC ETHICS REVIEW COMMITTEE
IRB REGISTRATION NUMBER : 00002911
FWA NUMBER : 00003729

TRC/C4/07/ 2021

23rd October 2021

Dr. Severine Nyerembe Anthony
Copperbelt University
NDOLA

Dear Dr. Nyerembe

RE: RENEWAL OF ETHICAL APPROVAL OF STUDY PROTOCOL

Reference is made to the protocol entitled **"EFFECTIVENESS OF MODIFIED BASIC PACKAGE OF ORAL CARE ON ORAL HEALTH STATUS AND RELATED QUALITY OF LIFE AMONG ZAMBIAN ADOLESCENTS-: A FIELD TRIAL"** and your request for further amendments to your protocol.

On behalf of the Chairperson of the TDRC Research Ethics Committee (REC), I wish to inform you that the Committee, reviewed your study progress report and considered your request for renewal of ethics approval for the above study.

The Committee has taken note of the progress and the milestones attained this far. To this effect, the Committee has no objection to extending ethics approval for this study to **23rd October, 2022**

We wish you success in the execution of the study.

Yours faithfully
TROPICAL DISEASES RESEARCH CENTRE

23 OCT 2021

Edna Mwale, Simbayi
SECRETARY – TDRC Ethics Review Committee

cc: Chairperson – TDRC Ethics Review Committee

TROPICAL DISEASES RESEARCH CENTRE
Tel/Fax +260212 615444
P O Box 71769
tdrc-ethics@tdrc.org.zm
NDOLA, ZAMBIA

TDRC ETHICS REVIEW COMMITTEE
IRB REGISTRATION NUMBER : 00002911
FWA NUMBER : 00003729

TRC/C4/07/ 2022

23rd October 2022

Dr. Severine Nyerembe Anthony
Copperbelt University
NDOLA

Dear Dr. Nyerembe

RE: RENEWAL OF ETHICAL APPROVAL OF STUDY PROTOCOL

Reference is made to the protocol entitled **"EFFECTIVENESS OF MODIFIED BASIC PACKAGE OF ORAL CARE ON ORAL HEALTH STATUS AND RELATED QUALITY OF LIFE AMONG ZAMBIAN ADOLESCENTS-: A FIELD TRIAL"** and your request for further amendments to your protocol.

On behalf of the Chairperson of the TDRC Research Ethics Committee (REC), I wish to inform you that the Committee, reviewed your study progress report and considered your request for renewal of ethics approval for the above study.

The Committee has taken note of the progress and the milestones attained this far. To this effect, the Committee has no objection to extending ethics approval for this study to **23rd October, 2023**

We wish you success in the execution of the study.

Yours faithfully
TROPICAL DISEASES RESEARCH CENTRE

23 OCT 2022

Edna Mwale. Simbayi
SECRETARY – TDRC Ethics Review Committee
cc: Chairperson – TDRC Ethics Review Committee

TROPICAL DISEASES **RESEARCH CENTRE**
Tel/Fax +260212 615444
P O Box 71769
tdrc-ethics@tdrc.org.zm
NDOLA, ZAMBIA

TDRC ETHICS REVIEW COMMITTEE
IRB REGISTRATION NUMBER : 00002911
FWA NUMBER : 00003729

TRC/C4/07/ 2023

23rd October 2023

Dr. Severine Nyerembe Anthony
Copperbelt University
NDOLA

Dear Dr. Nyerembe

RE: RENEWAL OF ETHICAL APPROVAL OF STUDY PROTOCOL

Reference is made to the protocol entitled **"Effectiveness of Modified Basic Package of Oral Care On Oral Health Status and Related Quality of Life Among Zambian Adolescents-: A Field Trial"** and your request for further amendments to your protocol.

On behalf of the Chairperson of the TDRC Research Ethics Committee (REC), I wish to inform you that the Committee, reviewed your study progress report and considered your request for renewal of ethics approval for the above study.

The Committee has taken note of the progress and the milestones attained this far. To this effect, the Committee has no objection to extending ethics approval for this study to **23rd October, 2024**

We wish you success in the execution of the study.

Yours faithfully
TROPICAL DISEASES RESEARCH CENTRE

Edna Mwale. Simbayi
SECRETARY – TDRC Ethics Review Committee

23 OCT 2023

cc: Chairperson – TDRC Ethics Review Committee

MUHIMBILI UNIVERSITY OF HEALTH AND ALLIED SCIENCES

OFFICE OF THE DIRECTOR OF RESEARCH AND PUBLICATIONS

P.O. Box 65001
DAR ES SALAAM
TANZANIA
Web: www.muhas.ac.tz

Tel G/Line: +255-22-2150302/6 Ext: 1016
Direct Line: +255-22-2152489
Telefax: +255-22-2152489
E-mail: drp@muhas.ac.tz

Ref. No.DA.282/298/01.C/ 21st July, 2020

P.MUHAS-REC-4-2020-208

Dr. Severine Nyerembe Anthony,
Department of Orthodontics Paedodontics & Community Dentistry,
School of Dentistry,
MUHAS.

RE: APPROVAL FOR ETHICAL CLEARANCE FOR A STUDY TITLED "EFFECTIVENESS OF MODIFIED BASIC PACKAGE OF ORAL CARE ON ORAL HEALTH STATUS AND RELATED QUALITY OF LIFE AMONG ZAMBIAN ADOLESCENTS:- A FIELD TRIAL"

Reference is made to the above heading.

I am pleased to inform you that the Chairman has on behalf of the University Senate, approved ethical clearance of the above mentioned study, on recommendations of the Senate Research and Publications Committee Meeting.

The validity of this ethical clearance is one year effective from to **24th June, 2020 23rd June, 2021.** You will therefore be required to apply for renewal of ethical clearance on a yearly basis if the study is not completed at the end of this clearance.

You will be expected to provide adverse events report where applicable, six monthly progress reports and a final project report upon completion of your study.

Dr. Bruno Sunguya
Chairman, MUHAS Research and Ethics Committee

Region:	Saksbehandler:	Telefon:	Vår dato:	Vår referanse:
REK vest	Fredrik Kolstad Rongved	55978498	29.12.2020	191836
			Deres referanse:	

Anne Nordrehaug Åstrøm

191836 Effectiveness of modified basic package of oral care on oral health status and related quality of life among Zambian adolescents- a field trial

Forskningsansvarlig: Muhimbili University of Health and Allied Sciences

Søker: Anne Nordrehaug Åstrøm

Søkers beskrivelse av formål:

Background: Global trends of diseases show an increase of oral diseases in most low and middle- income countries due to increased availability of sugars, inadequate fluoride products and lack of sustainable preventive strategies. Most oral diseases are behavioral related and behaviors acquired during adolescence are likely to be passed to adulthood. Adolescent's oral health behaviors are shaped by family socioeconomic status and therefore socio gradient in oral diseases may be more reflected in this group. Despite increasing oral diseases and Word Health Organization (WHO) recommendations of using BPOC as feasible and cost-effective preventive and curative approach of oral diseases, the strategy has not been implemented in Sub-Saharan Africa region. Aim: To evaluate the effectiveness of applying the modified WHO basic package of oral care (BPOC) in improving oral health-knowledge and behaviors, oral health and related quality of life among adolescents in Copperbelt, Zambia. Materials and methods: A cluster randomized controlled field trial with a total sample size of 1760 grade eight secondary school adolescents will be done in Copperbelt province, Zambia. Adolescents in the trial arm will receive a modified WHO-BPOC and the control will maintain their standard of care, both groups will be re-assessed at follow-ups. Data collection on adolescents' demographics, oral health-related knowledge and behavior, impacts of oral disease on daily performance and parental socio-demographics will be done using a self-administered structured questionnaire. Data on dentition status, plaque, and gingival bleeding will be assessed using CAST, Silness, and Loe (1967) plaque and gingival bleeding indices, respectively. Data entry, cleaning, and analysis will be done using IBM SPSS for windows (version 23), and summarized as frequency distribution and cross-tabulations. Adjusted binary logistic regression and generalized linear model will be performed to examine the differences between the groups at baseline and follow up in oral health-related behaviors, plaque, gingival bleeding, OHRQoL and dental caries status for various socio-demographics characteristics. The statistical significance difference shall be assumed when p 0.05.

REKs vurdering

We refer to the response to remarks by the Committee on the above-mentioned research project, received the 17th of November 2020. The application was assessed by the Chair of REC West on the authority of the Committee. The assessment was made pursuant to the Health Research Act § 10.

Requested feedback:

REC West requested a written statement justifying the need for this study. The Committee also had some remarks to the informed consent sheet.

Feedback:

The applicant has given feedback on the remarks by the Committee. Regarding the justification of the need for this study:

" Oral health services in Zambia have been inclined towards curative rather than preventive approach for number of decades due to lack of emphasis, planning, budgeting and funding of preventive services. The curative approach relies on inadequate number and unevenly distributed dental clinics which are mainly concentrated in urban areas. Most of the clinics render limited services mainly extraction of teeth, due to lack of enough funds to procure equipment and materials for restorative services. Accessibility and affordability of available curative services is a challenge especially among rural communities which may potentially create oral health inequalities. Word Health Organization (WHO) recommended Basic Package of Oral Care (BPOC) as an easy to conduct, cheap, and effective strategy in prevention and management of oral diseases in low to middle income countries. Despite of potential benefits the strategy has not been tested in most countries including Zambia. The shortfall could be explained by inadequate advocacy of importance of preventive over curative oral health services during training and oral health delivery, inadequate planning and budgeting for oral health research and preventive services, and lack of oral health data compared to other NCDs in some African countries including Zambia. One of the targets of 2017-2021 Zambia National Health strategic plan is to strengthen and orient health systems to address the prevention and control of NCDs and the underlying social determinants through people-centered primary health care and Universal Health Coverage. This goal cannot be realized without evidence based results of the magnitude of non-communicable disease in particular oral diseases and effectiveness of preventive strategies such as WHO-BPOC. This study will highlight the magnitude of dental caries and gingivitis among adolescents and effectiveness of WHO-BPOC strategy in oral diseases prevention. The data will help oral health planners to convince policy makers and planners at ministry of health to plan for preventive oral health services in the Country. The study will also improve training of the fifty-year bachelor of dental surgery students who will be involved during execution of BPOC components (OUT, AFT and ART) under supervision. The training will not only impart knowledge on prevention to the dental students but also orient them towards diseases prevention. The study will also contribute towards realizing the 2017-2021 strategic plan goal of creating sustainable and peoples –centered primary health care by empowering students and teachers assigned to the project with knowledge to give oral health education."

The text of a revised informed consent sheet has been added in the feedback.

Local ethics approvals:

REC received the ethical approvals from Zambia and Tanzania the 18th of December.

Assessment:

REC west considers the response to be satisfactory and have no further remarks.

Vedtak

Godkjent

REC west have made a narrow research ethics assessment of the project. The project is approved pursuant to the Health Research Act § 10.

Sincerely
Marit Grønning
Prof. dr. med.
Chair of REC West

Fredrik Rongved
advisor

Sluttmelding
Søker skal sende sluttmelding til REK vest på eget skjema senest seks måneder etter godkjenningsperioden er utløpt, jf. hfl. § 12. Dersom prosjektet ikke igangsettes eller gjennomføres skal prosjektleder også sende melding om dette via sluttmeldingsskjemaet.

Søknad om å foreta vesentlige endringer
Dersom man ønsker å foreta vesentlige endringer i forhold til formål, metode, tidsløp eller organisering, skal søknad sendes til den regionale komiteen for medisinsk og helsefaglig forskningsetikk som har gitt forhåndsgodkjenning. Søknaden skal beskrive hvilke endringer som ønskes foretatt og begrunnelsen for disse, jf. hfl. § 11.

NATIONAL HEALTH RESEARCH AUTHORITY
Paediatric Centre of Excellence, University Teaching Hospital, P.O. Box 30075, LUSAKA
Tell: +260211 250309 | Email: znhrasec@gmail.com | www.nhra.org.zm

Ref No: NHRA00005/16/11/2020 **Date: 16th November, 2020**

The Principal Investigator
Dr. Severine Nyerembe Anthony,
Copperbelt University,
Michael Chilufya Sata – School of Medicine,
Ndola.

Dear Dr. Nyerembe,

Re: Request for Authority to Conduct Research

The National Health Research Authority is in receipt of your request for authority to conduct research titled **"EFFECTIVENESS OF MODIFIED BASIC PACKAGE OF ORAL CARE ON ORAL HEALTH STATUS AND RELATED QUALITY OF LIFE AMONG ZAMBIAN ADOLESCENTS-: A FIELD TRIAL."** I wish to inform you that following submission of your request to the Authority, our review of the same and in view of the ethical clearance, this study has been **approved** on condition that:

1. The relevant Provincial and District Medical Officers where the study is being conducted are fully appraised;
2. Progress updates are provided to NHRA quarterly from the date of commencement of the study;
3. The final study report is cleared by the NHRA before any publication or dissemination within or outside the country;
4. After clearance for publication or dissemination by the NHRA, the final study report is shared with all relevant Provincial and District Directors of Health where the study was being conducted, University leadership, and all key respondents.

Yours sincerely,

Prof. Godfrey Biemba
Director/CEO
National Health Research Authority
